# Versatile, modular, and customizable magnetic solid-droplet systems

**DOI:** 10.1073/pnas.2405095121

**Published:** 2024-08-01

**Authors:** Mengmeng Sun, Yingdan Wu, Jianhua Zhang, Hongchuan Zhang, Zemin Liu, Mingtong Li, Chunxiang Wang, Metin Sitti

**Affiliations:** ^a^Physical Intelligence Department, Max Planck Institute for Intelligent Systems, Stuttgart 70569, Germany; ^b^State Key Laboratory of Robotics and Systems, Harbin Institute of Technology, Harbin 150001, China; ^c^School of Medicine and College of Engineering, Koç University, Istanbul 34450, Türkiye

**Keywords:** soft robotics, ferrofluid, solid-droplet systems, magnetically driven

## Abstract

Magnetic miniature robotic systems have attracted broad research interest because of their precise maneuverability in confined spaces and adaptability to diverse environments, holding significant promise for applications in both industrial infrastructures and biomedical fields. However, the predominant construction methodology involves the preprogramming of magnetic components into the system’s structure. While this approach allows for intricate shape transformations, it exhibits limited flexibility in terms of reconfiguration and presents challenges when adapting to diverse materials, combining, and decoupling multiple functionalities. Here, we propose a construction strategy that facilitates the on-demand assembly of magnetic components, integrating ferrofluid droplets with the system’s structural body. This approach enables the creation of complex solid-droplet robotic systems across a spectrum of length scales, ranging from 0.8 mm to 1.5 cm. It offers a diverse selection of materials and structural configurations, akin to assembling components like building blocks, thus allowing for the seamless integration of various functionalities. Moreover, it incorporates decoupling mechanisms to enable selective control over multiple functions, leveraging the fluidity, fission/fusion, and magneto-responsiveness properties inherent in the ferrofluid. Various solid-droplet systems have validated the feasibility of this strategy. This study advances the complexity and functionality achievable in small-scale magnetic robots, augmenting their potential for future biomedical and other applications.

## Significance

Wireless miniature multifunctional robots hold promise for industrial and biomedical applications, yet coordinating their diverse functional modules poses a significant challenge. We propose a versatile, modular, and customizable magnetic solid-droplet system, combining magnetic ferrofluid droplets within solid-shell functional modules. The ferrofluid acts as the trigger, with the modular solid shells providing various functions. These modules can be effortlessly assembled, allowing the ferrofluid droplets to move through connecting channels, selectively triggering different modules as needed. This approach facilitates coordinated real-time actuation of different functionalities within the system, making it ideal for complex procedures requiring sequential or simultaneous actions particularly in medical applications.

Wirelessly powered miniature robots, responsive to external stimuli (1–8), exhibit compactness, minimal invasiveness characteristics, and offer transformative applications across diverse fields, especially in biomedicine (9–14). Notably, wireless magnetic miniature robots present distinct advantages regarding safety, speed, precision, and versatility in their response (15–19). Recent advances have demonstrated the versatility of magnetically actuated small-scale robots, encompassing a broad spectrum of motion patterns (20–22), variable morphologies (23–27), and multifunctionality (28–32). Applications range from cell transplantation to drug delivery, sampling, and more (33–39). Fabrication methods for magnetic miniature robots encompass various techniques such as molding and casting (16, 17, 20, 22, 23, 26), additive manufacturing (2, 15, 19, 24), microfabrication (28, 30), and coating (18, 29, 33, 34, 39). While the manufacturing methods may vary, the fundamental construction principles remain consistent, involving the preprogramming of magnetic components into the bodies or surfaces of miniature devices (15, 40–42). Consequently, these magnetic elements are fixed and cannot be reconfigured with respect to the structural elements of the devices as required. This basic construction principle gives rise to several limitations associated with the current preparation method, encompassing restricted material choices, challenges related to biocompatibility, single functionality, and the inability to decouple multiple functions.

Specifically, elastomers and gels are commonly used as matrix materials in the conventional construction method for embedding magnetic components, such as micro/nanoparticles, in a fixed configuration (19–32). This rigidity highlights several issues with the magnetic components, including global responsiveness, cytotoxicity, nondegradability, corrosion, and design constraints. First, such fixed embedding within the robot’s body causes each part to respond uniformly to external magnetic fields, limiting selective or independent control of different robot components. This uniform responsiveness hampers the integration of multiple functionalities, crucial for complex diagnostic and therapeutic tasks that require coordinative execution of sampling and injection functions in biomedical applications. First, such fixed embedding within the robot’s body causes each part to respond uniformly to external magnetic fields, limiting selective or independent control of different robot components. This uniform responsiveness hampers the integration of multiple functionalities, crucial for complex diagnostic and therapeutic tasks that require coordinative execution of sampling and injection functions in biomedical applications (15). In addition, the conventional method of embedding magnetic components in matrix polymers is in favor of using hard ferromagnetic particles like Neodymium Iron Boron (NdFeB) to achieve substantial deformation under external magnetic control. Despite the inherent softness and flexibility of these polymers (43–45), the use of hard particles poses significant cytotoxic risks due to their corrosive nature (46), limiting their applications in biomedical scenarios. Although alternative approaches such as using biocompatible magnetic components (32) or applying biocompatible coatings (19) have been explored, these methods do not adequately address the core issues. The fixed embedding technique remains unsuitable for biomedical devices intended for long-term use or those requiring biodegradability, such as tissue scaffolds and implantable (33, 34) or cell transplantation devices (47). This is due to the risks of leakage and corrosion (15) and challenges in retrieving nondegradable particles (47, 48). Moreover, the embedding of magnetic components can impact various properties of these matrix materials, such as mechanical, physicochemical, and other performance characteristics; thus, requiring strict control over the content of magnetic elements (15, 43). All these constraints limit the design space and the range of functions achievable in current small-scale devices. Consequently, they hinder the practical realization of theoretically promising designs or induce compromises in functionality.

**Fig. 1.**
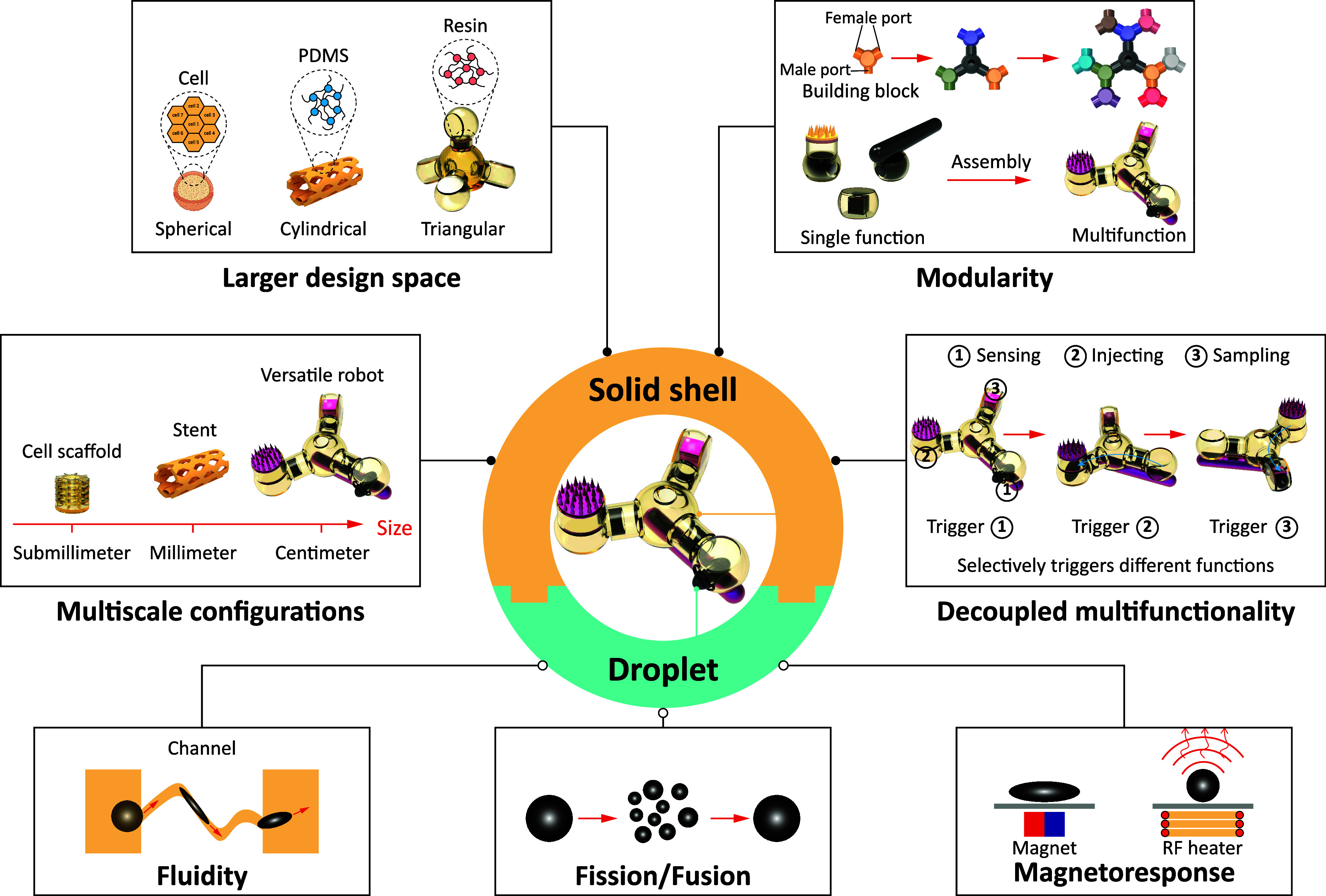
The concept of solid-droplet systems. Unlike existing magnetic miniature robots, which typically incorporate magnetic drive components (such as magnetic nanoparticles) into the robot’s structure or surface coating, solid-droplet systems combine ferrofluids with external solid shells. In this design strategy, ferrofluids serve as magnetic field drive components, while the outer solid shell confines the ferrofluid and plays a vital functional role. Ferrofluids possess remarkable properties, including fluidity that allows them to traverse narrow channels and be separated from the outer shell. When exposed to a magnetic field, they can undergo extreme deformations, such as splitting and fusing. Moreover, ferrofluids exhibit exceptional responsiveness to magnetic fields, capable of movement under quasi-static magnetic fields and generating heat when subjected to an external radio frequency (RF) field. One of the key advantages of this approach is its adaptability. By varying the dimensions of the solid shell, solid-droplet systems can be created in a range of sizes, from submillimeters to centimeters. Furthermore, altering the shape and material composition of the outer shell expands the design space, enabling the creation of robots with diverse configurations and functions, such as cell scaffolds or stents. To harness the full potential of solid-droplet systems, various parts are standardized into functional modules, akin to assembling other solid-droplet systems using Lego blocks. Different function modules can also be selectively activated by driving ferrofluids to various positions. Consequently, this design philosophy offers unparalleled customization and versatility when assembling multifunctional miniature robots.

We propose a construction strategy to address these challenges by incorporating reconfigurable magnetic components into the structural elements of the device. This approach yields various miniature solid-droplet systems, which leverage the properties of magnetic ferrofluid droplets, including fluidity, fusion/fission capability, and magneto-responsiveness (18, 21, 29, 38, 49–51), and is compatible with solid shells of varying sizes, structures, and compositions (Fig. 1). Initially, we elucidate the mechanism underlying the reconfigurable assembly and disassembly of ferrofluid droplets with the solid outer shell. We establish design principles and actuation strategies for solid-droplet systems, and then characterize the factors influencing the coupling between the ferrofluid droplets and the host material. Subsequently, combining the ferrofluid droplets with the outer shells of different materials can create cargo delivery platforms; e.g., the proposed solid-droplet devices can transport cell spheroid shells, scaffolds, or stent-shaped structures. Additionally, the magneto-thermal properties of ferrofluid droplets enable the actuation of thermally responsive modules. The ferrofluid droplets can also encapsulate liquid or solid payloads, facilitating precise delivery or sampling while preserving the integrity of the enclosed materials. Based on these properties, we demonstrate exemplary functional modules for our solid-droplet systems, including a thermally triggered microneedle patch, a wireless sensor carrier with controllable deployment capabilities, and a liquid biopsy device designed for both contamination resilience and access to challenging-to-reach sites. By standardizing these functions as modular components, they can be seamlessly pieced together, akin to assembling LEGO blocks, to create multifunctional assemblies. The inherent fluidity and fission/fusion properties of ferrofluid droplets enable the selective control within multifunctional assemblies, allowing for either sequential triggering or the simultaneous actuation of multiple functional modules as needed. Therefore, this approach expands the design space, enhances the system’s functionality, and provides a tool to develop wireless functional miniature devices tailored to complex biomedical applications.

## Results

### Working Principles of Solid-Droplet Systems: Assembly, Actuation, and Separation.

**Fig. 2.**
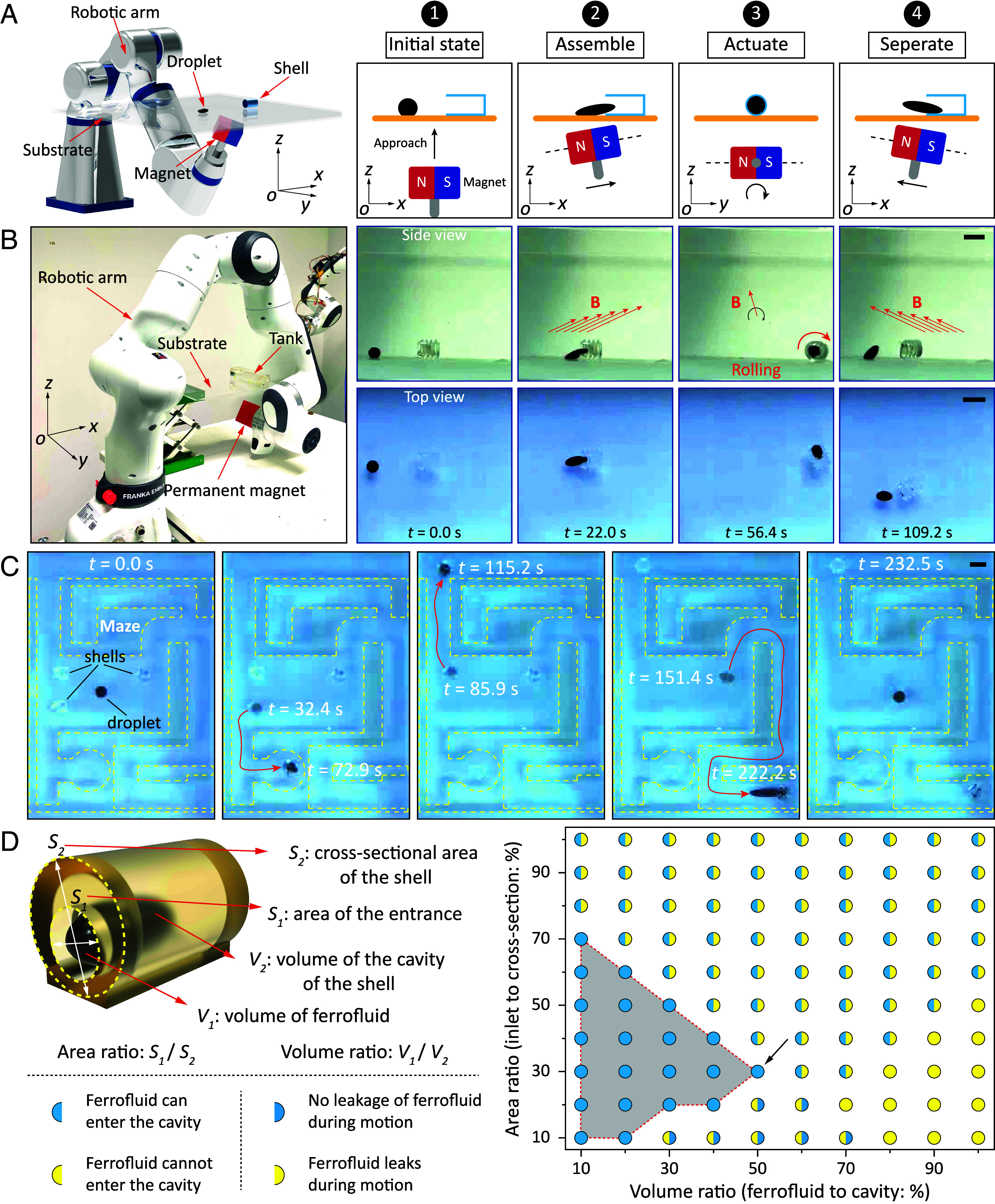
Mechanisms for assembling, actuating, and separating ferrofluids with solid shells. (*A*) Schematic showing the assembly, actuation, and separation of ferrofluid droplets and solid shells under a magnetically controlled system consisting of a robotic arm and a permanent magnet. This setup is used to realize the assembly, locomotion, and separation of droplets with different shells. (*B*) Snapshots show a ferrofluid droplet of 2.5 mm diameter is assembled with a helical structured shell of 3 mm length driven by a magnetically controlled system, carries it in motion, and then separates from it. (*C*) Snapshots of a ferrofluid droplet assembled sequentially with three solid shells, transported to different target locations, and separated from them. (*D*) Effect of volume and area ratios on the assembly of magnetic fluid droplets and solid shells. The shaded area indicates that the droplet can easily enter the solid shell and will not leak. The arrow indicates the optimal solution. (Scale bar, 3 mm.)

Our proposed solid-droplet system consists of two key components: the reconfigurable magnetic component, i.e., the ferrofluid droplet, and the nonmagnetic solid outer shell, which encases the ferrofluid droplet, providing a platform for various functionalities. The schematic in Fig. 2*A* illustrates the active control of the ferrofluid droplet in millimeter-sized shells using an external gradient magnetic field to enter and leave through the shell’s opening and interact with the body, forming a solid-droplet system. This process consists of three main steps: assembly, actuation, and separation. As shown in Fig. 2*B*, the cylindrical resin shell features an opening on one end, with a maximum diameter of 3 mm. The inward-oriented gradient magnetic field drives the ferrofluid droplet with a diameter of 2.5 mm to enter the shell. The ferrofluid then adheres to the inner wall of the shell and moves the body in a controlled manner via the external magnetic field (Movie S1). The difference in the solid shell’s shape also affects the solid-droplet system’s motion mode. A helical shell facilitates rolling motion when subjected to a rotating magnetic field, while a hexahedral shell, less suited for rolling, can be pulled into motion via a gradient magnetic field (*SI Appendix*, Fig. S1). Once the assembled solid-droplet system reaches the targeted position, the ferrofluid can either activate the shell’s function or detach from it by applying an outward-oriented gradient magnetic field. The advantage of active assembly lies in its ability to achieve reversible, in situ construction of solid-droplet systems and perform multiple reconfigurations of the whole system as required by the task.

The separability of the ferrofluid droplet from the body enables it to drive different shell modules selectively. Fig. 2*C* shows that the ferrofluid sequentially transports three shells to different targeted locations in a maze (Movie S2). Realizing this selective control of different magnetic robots is challenging because they all react to the global magnetic field. However, since the magnetic ferrofluid droplet can be reversibly separated and assembled with each shell, it can move each shell independently. The ferrofluid droplet separates from the shell at the targeted position, allowing it to combine with other shells and sequentially transport them to their respective targeted positions. This selective control ability broadens the potential applications of wireless magnetic robots. To facilitate the assembly process and enhance the system mobility, we examine two key design factors of the system: the droplet volume-to-shell volume ratio (volume ratio) and the entrance area-to-cross-sectional area ratio (area ratio). A higher area ratio simplifies assembly but may lead to leakage during motion. A larger volume ratio provides more propelling force but has an encasement limit. Striking a balance between these ratios is key for system optimization. We thoroughly investigate the effect of these ratios on the operation of the solid-droplet systems (Fig. 2*D*). The results indicate that when the volume ratio ranges from 10 to 50%, and the area ratio ranges from 10 to 70%, denoted as a shaded area in Fig. 2*D*, the solid-droplet systems are easy to be assembled and less prone to leakage during motion. The optimal parameters are a 30% area ratio and a 50% volume ratio, improving both assembly ease and system mobility.

### Interactions of Ferrofluid Droplets with Different Solid Shell Surfaces.

**Fig. 3.**
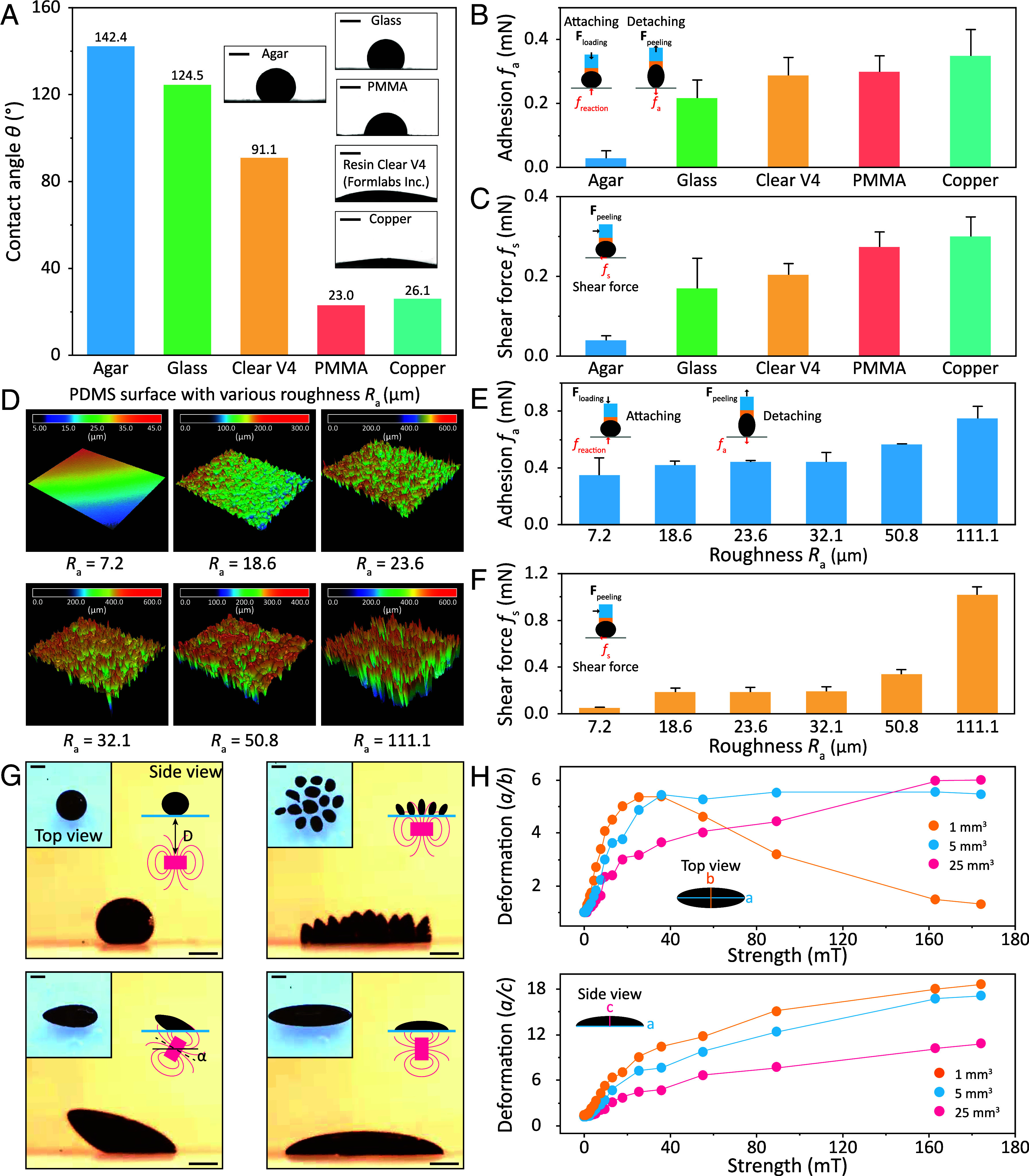
Quantitative assessment of ferrofluid droplet’s adhesion and shear forces on different surfaces, as well as their deformability under the influence of various magnetic fields. (*A*) Contact angle tests of ferrofluids on different material surfaces. The inset figures show the contact angle of ferrofluid droplets on different surfaces. The adhesion (*B*) and shear force (*C*) measurement of ferrofluid droplet on different solid substrates with the same roughness (about 10 µm) under preload of 3 mN. (*D*) The laser-optical scanning images of the PDMS surface with different roughness. The adhesion (*E*) and shear force (*F*) measurement of ferrofluid droplets on the same solid substrates (PDMS) with different roughness under preload of 3 mN. (*G*) Magnetic field-induced deformation of a ferrofluid droplet in immiscible deionized water. The *Top* and side views of the ferrofluid droplet show that the magnet’s different positions (*D* and *α*) make its deformation morphologically different. (*H*) The deformation of ferrofluid droplets with different volumes as a function of magnetic field strengths. In all figures, error bars indicate the SD for *n* = 5 measurements. (Scale bar, 1 mm.)

We quantitatively analyze the wetting dynamics, adhesion, and shear forces between ferrofluid droplets and various material surfaces to effectively implement ferrofluid droplets in solid-droplet systems. We use the contact angle as a quantifiable metric to assess the wetting dynamics of ferrofluid droplets on different substrate surfaces. Fig. 3*A* compares the contact angles of ferrofluid droplets on agar, glass, photo resin Clear V4 (Formlabs Inc.), polymethyl methacrylate (PMMA), and copper surfaces without an external magnetic field. The measured contact angles are 142.4°, 124.5°, 91.1°, 23.0°, and 26.1°, respectively, while the surface roughness of these materials averages around 10 µm. These variations in contact angles are attributed to different materials’ distinct hydrophilic and hydrophobic properties, which also influence adhesion and shear forces between ferrofluid droplets and the surfaces. Adhesion and shear forces are pivotal in constructing solid-droplet systems, as they dictate ferrofluid assembly, movement, and separation from the shell. Fig. 3 *B* and *C* present a comparison of adhesion and shear forces between ferrofluid droplets and diverse material surfaces (the characterization protocol for adhesion and shear forces is detailed in *SI Appendix*, Fig. S2). Notably, surfaces with higher hydrophilicity, characterized by larger contact angles, exhibit reduced adhesion and shear forces when interacting with ferrofluid droplets. For instance, the adhesion and shear forces between ferrofluid droplets and photo resin Clear V4 material measure 0.028 mN and 0.038 mN, respectively. In contrast, the adhesion and shear forces between ferrofluid droplets and copper material are recorded at 0.348 mN and 0.299 mN (*SI Appendix*, Figs. S3 and S4). Consequently, modifying the hydrophilic or hydrophobic properties of surfaces can be employed to tune the adhesion and shear forces between ferrofluid droplets and the shell surfaces. The roughness (*R_a_*) of shell surfaces also plays a significant role in their interaction with ferrofluid droplets. We examine the adhesion and shear forces between ferrofluid droplets and polydimethylsiloxane (PDMS) surfaces with various roughness levels, ranging from 7.2 to 111.1 µm (Fig. 3*D* and *SI Appendix*, Fig. S5). Lower surface roughness (*R_a_* = 7.2 µm) results in reduced adhesion and shear forces with ferrofluid droplets (0.347 mN, 0.050 mN, respectively). Conversely, higher surface roughness (*R_a_* = 111.1 µm) increases adhesion and shear forces with ferrofluid droplets (0.751 mN, 1.101 mN, respectively). Fig. 3 *E* and *F* show the positive correlation between adhesion, shear forces, and surface roughness. This justifies the feasibility of tuning the inner wall’s roughness of the shell to control the interaction between the ferrofluid droplet and the shell.

With their unique combination of liquid properties and robust magnetic responsiveness, ferrofluid droplets exhibit diverse and fascinating functional characteristics. Notably, ferrofluid droplets can undergo intriguing ferrohydrodynamic instabilities. These instabilities, influenced by factors such as external magnetic fields, result in abrupt changes in the configuration of ferrofluid droplets. Such variations in ferrofluid droplet configuration are pivotal in solid-droplet systems. When ferrofluid droplets actively interact with the shell, they need to swiftly adapt to deformations in the inlet direction to ensure effective entry into the body. Additionally, as the ferrofluid droplet transports the shell, it must preserve the overall structural integrity of the assembly, preventing any undesired separation. Furthermore, when the solid-droplet system is required to perform multiple functions simultaneously, the ferrofluid droplet should demonstrate controlled division to activate those modules concurrently. Last, upon separation from the shell, the ferrofluid droplet should deform along the outlet direction to achieve detachment from the body. To gain insights into these morphological transformations, we conducted experiments to explore the morphological configurations of oil-based ferrofluid droplets within an immiscible solvent, such as deionized water. We apply external magnetic fields with varying strength and orientation by adjusting the position of the permanent magnet beneath (the relationship between the distance from the permanent magnet and the magnetic field strength is shown in *SI Appendix*, Fig. S6). As depicted in Fig. 3*G*, when the permanent magnet approaches the ferrofluid droplet positioned on agar from various vertical distances, the droplet enters an unstable state marked by a critical field strength of 160 mT. This instability leads the morphology of the droplet to transition from a spherical whole into multiple subdroplets. Upon changing the angle of the permanent magnet from vertical to horizontal gradually, the ferrofluid transforms into a shuttle-like configuration. Notably, the long axis of the shuttle-shaped ferrofluid droplet aligns with the direction of the permanent magnet. We quantitatively evaluate the length-to-width and length-to-height ratios of the ferrofluid droplet in the horizontal shuttle state as functions of magnetic field strengths. Fig. 3*H* illustrates that the observed trends are not consistent across different volumes of ferrofluid. For small-volume ferrofluid droplets (<1 mm^3^), the length-to-width ratio initially increases, reaching its peak at 5.456 with a magnetic field strength of 28 mT, and then decreases to 1.370 as the strength increases up to 160 mT. In contrast, for large-volume ferrofluid droplets (>5 mm^3^), the length-to-width ratio increases and stabilizes with the increasing magnetic field strength. However, it’s noteworthy that the length-to-height ratio consistently increases with growing magnetic field strength for both small-volume and large-volume droplets. Based on the above conclusions we can choose a suitable magnetic field configuration to control the morphology of the ferrofluid droplets in order to drive the solid-droplet system.

### Magnetic Solid-Droplet Systems for Cargo Delivery.

**Fig. 4.**
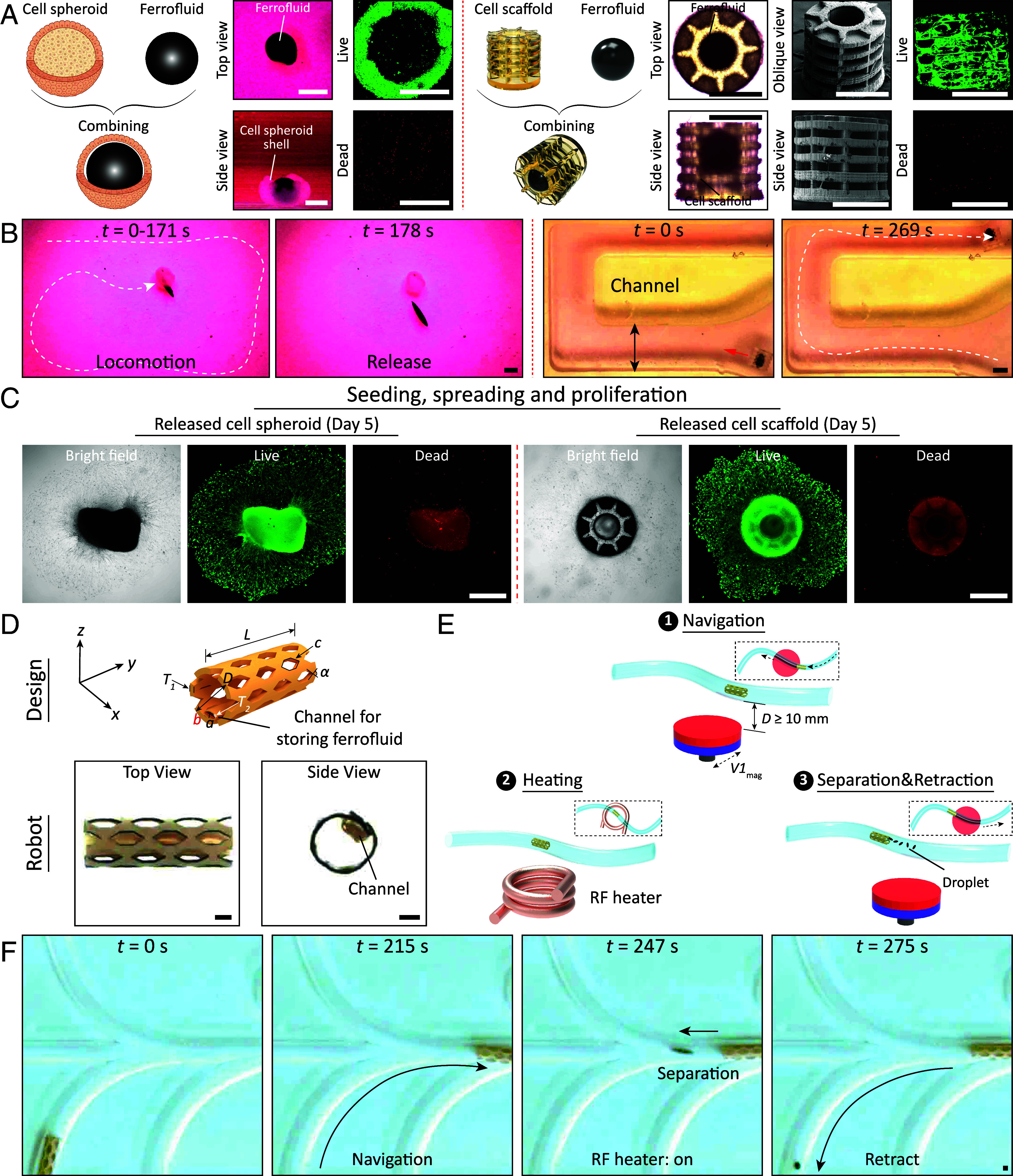
Magnetically actuated solid-droplet systems for cargo delivery. (*A*) Conceptual drawing showing the assembly process of ferrofluid droplets with a cell spheroid shell, forming a cell spheroid shell robot, and with a cell scaffold, resulting in a cell scaffold robot. The *Insets* demonstrate the viability of the cell spheroid shell and cell scaffold, indicated by live cells in green and dead cells in red. Additionally, scanning electron microscopy (SEM) images reveal the structural details of the fabricated cylindrical cell scaffold. (*B*) Assembled cell spheroid shell robot and cell scaffold robot, actuated by a magnetic field, and subsequently released. (*C*) The released cell spheroid shells and scaffolds can seed, spread, and proliferate on the substrate. (*D*) Design drawing and the photo of the stent robot prototype. This stent robot comprises an external stent-shaped structure and an internal ferrofluid storage cavity. Key design parameters include length (*L*) = 9 mm, outer diameter (*D*) = 3 mm, thickness of the outer structure (*T*_1_) = 0.1 mm, length of hollow segments (*c*) = 1 mm, and angle (*α*) = 60°. The cavity for ferrofluid storage is sealed at both ends with thermal adhesive, employing gelatin with an inner diameter (*a*) = (*b*) = 1 mm and a thickness (*T*_2_) = 0.05 mm. (*E*) Overall concept of the stent robot’s delivery process in tortuous routes, involving navigation, heating, separation, and retraction. During navigation, a magnetic field gradient force propels the stent robot. Upon reaching the target position, heat generated by the ferrofluid droplet melts the thermal adhesive, activated by RF fields. Subsequently, driven by the magnetic field, the ferrofluid droplet separates from the cavity and withdraws from the target position. (*F*) The stent robot’s movement through tortuous channels to the target position, achieving solid-droplet separation and facilitating the release of the stent to withdraw the ferrofluid droplets. In all figures, error bars indicate the SD for *n* = 3 measurements. [Scale bar, 500 µm (*A*–*C*), 1 mm (*D* and *F*).]

Regenerative medicine, particularly cell-based therapy, has garnered significant attention recently (52). To successfully deliver these cells in vivo, a three-dimensional (3D) structure is necessary as a platform supporting cell adhesion, proliferation, and differentiation. Microrobots actuated by magnetic forces have emerged as valuable tools for enabling cell transplantation in the intricate and confined regions of the human body (33, 34). While various magnetic-driven microrobots have been reported for cell delivery, existing one’s present challenges related to degradation, limiting their applications in long-term cell therapy scenarios. Researchers have also developed magnetic cell spheroid microrobots through the 3D self-assembly of stem cells (35). However, these microrobots exhibit weak magnetic properties, as they can only be doped with biocompatible, yet weak magnetic particles at relatively low concentrations. Considering this, we employ our solid-droplet system to assemble ferrofluid droplets with cell spheroid shells or scaffolds, constructing a platform for cell transplantation. The schematic and bright-field diagram in Fig. 4*A* illustrates the assembly of ferrofluid droplets with cell spheroid shells and scaffolds, respectively. The spheroid shell robot features a diameter of approximately 0.9 mm (*SI Appendix*, Fig. S7), with a ferrofluid droplet volume of roughly 0.5 mm^3^. The scaffold robot exhibits a diameter of about 800 µm, with a ferrofluid volume of approximately 0.08 mm^3^. Cell viability, as determined through cell dead-activity staining, demonstrated the sustained activity of the assembled cellular robots. *SI Appendix*, Fig. S8 assesses the impact of ferrofluids on cell viability, with the findings showing that ferrofluid volumes not exceeding 1.5 mm^3^ do not affect the viability of 1 mm-sized cell spheroid shells. Furthermore, the viability remains unaffected when ferrofluid contact with cell spheroid shells does not exceed 72 h. We investigate controlled locomotion, anchoring, seeding, and spreading of the spheroid shell and scaffold robots. As depicted in Fig. 4*B*, the spheroid shell and scaffold robots can be precisely navigated using the magnetic field, and it’s possible to control multiple cell spheroid shells (*SI Appendix*, Fig. S9). Upon reaching the targeted location, a strong gradient magnetic field separates the ferrofluid droplet from the robots, resulting in its release (Movie S3). Moreover, upon release, the cell spheroid shell and scaffold exhibit adaptive behavior, including seeding, spreading, and proliferation, resulting in their transformation into 2D configurations on cytophilic surfaces (Fig. 4*C*). This capacity holds significant promise for therapeutic applications within the body, as it enhances degradation processes. After 5 d, the cell spheroid and scaffold undergo spontaneous spreading and proliferation, transitioning from a 3D sphere to a 2D film-like structure through a straightforward incubation process at 37 °C. This transformation leads to a diffusion area approximately four times the initial cross-sectional area. Zoomed-in figures reveal that the cells within the cell spheroid and scaffold migrate outward, causing a shift in the configuration of cells at the outer ring from an irregular shape to a spreading form (*SI Appendix*, Fig. S10).

In addition to delivering cells, magnetic solid-droplet systems can also transport miniature medical devices, such as delivering stents to be placed in distal lumen regions. The existing magnetic stent robot has a body that contains toxic NdFeB magnetic particles despite the particles being coated with silicon dioxide on the surface to prevent rusting (16). However, stents prepared using this construction scheme still pose significant safety risks for prolonged in vivo applications. Therefore, we design and fabricate a cylindrical solid-droplet stent robot to address this challenge (Fig. 4*D* and *SI Appendix*, Fig. S11). The interior wall of the cylindrical stent structure features a sealed cavity by gelatin (10%), serving as a reservoir for ferrofluid droplets, allowing for their actuation. As depicted in Fig. 4*E*, the deployment of a stent robot encompasses four steps: navigation, heating, separation, and retraction. Fig. 4*F* presents the sequential processes involved in ferrofluid droplets’ movement, heating, and release from the stent robot (Movie S4). At *t* = 0 s, the stent robot navigates from the starting point. By *t* = 215 s, the stent robot successfully reaches the targeted position, upon which the radio frequency (RF) field (650 A, 338 kHz) is activated to initiate the heating process for subsequent release. The gelatin seals at both ends dissolve 32 s later (*t* = 247 s), releasing the ferrofluid droplet using an external magnetic field. *SI Appendix*, Fig. S12 demonstrates that ferrofluid droplets generate heat in the presence of an RF field (650 A, 338 kHz), accelerating gelatin dissolution. In the absence of heating, applying an external magnetic field does not result in leakage of ferrofluid droplets (*SI Appendix*, Fig. S13). Subsequently, the ferrofluid is retracted and returns to its starting point within 28 s (*t* = 275 s). The stent’s ability to navigate within phantoms featuring a continuous tortuous route, characterized by radii of curvature ranging from 2.5 mm to 4 mm, is demonstrated in *SI Appendix*, Fig. S14. In addition, we demonstrate that the stent robot can resist a flow rate of 12 mL/min against the flow and reaches its destination, and then eventually releases the droplets (*SI Appendix*, Fig. S15). It is worth noting that the design of the proposed stent is not constrained to a specific material. With the systematic experiments and modeling presented in this study, the system can be constructed using various Food and Drug Administration (FDA)-approved materials for medical devices. These materials may include polyurethane, polyethylene, and even metals.

### Magnetic Solid-Droplet Systems with Multiple Functions.

**Fig. 5.**
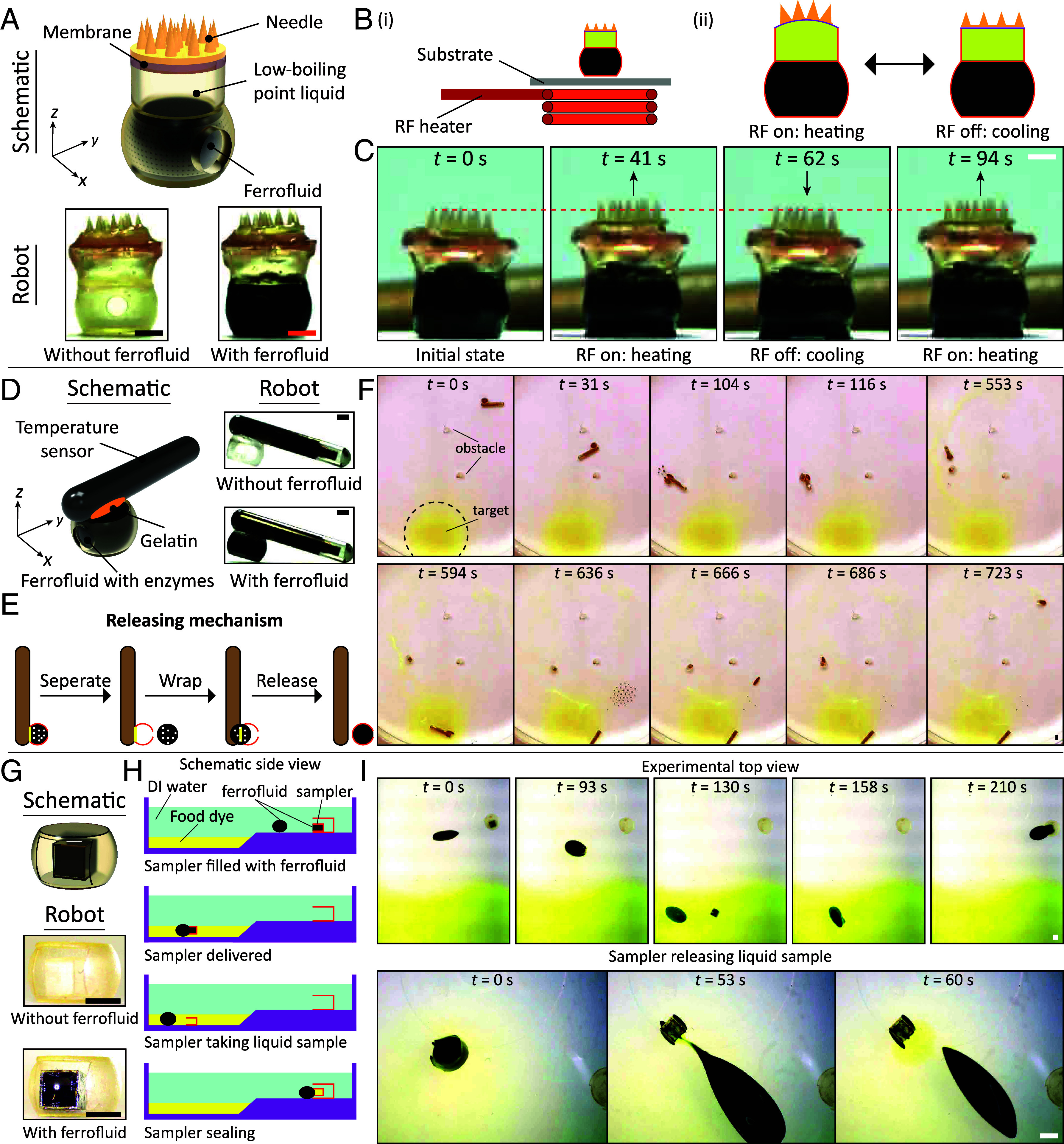
Magnetically actuated solid-droplet systems for microneedle patch, sensor delivery, and sampling liquid. (*A*) Design drawings of the microneedle patch robot and photos of the prototype. The microneedle patch robot consists of three parts: a microneedle patch array, a cavity for storing a low-boiling-point liquid (Novec 7000, the boiling point is ≈34 °C), and another cavity for storing ferrofluid droplet. (*B*) Schematic diagram of the working principle of the microneedle patch robot. The RF field induces the generation of heat in ferrofluid droplets, causing low-boiling-point liquids to boil, thereby expanding the storage chamber, ultimately resulting in the motion of the microneedle patch. (*C*) Snapshots of the up-and-down reciprocating motion of the microneedle patch robot in the air. The red dashed line indicates the initial position of microneedle patch in the off state of the external RF field. (*D*) Design drawings of the temperature sensor delivery robot and photos of the prototype. The sensor delivery robot consists of three elements: a miniature Radio-frequency identification (RFID) transponder with integrated temperature biosensor (UCT-2112, Unified Information Devices, USA), an adhesive substance (gelatin) for sensor fixation, and a cavity for storing ferrofluid droplet. (*E*) Schematic diagram of the temperature biosensor release principle by the sensor delivery robot. The ferrofluid droplet contains gelatin-dissolving enzymes, and when the sensor delivery robot reaches its intended location, the ferrofluid droplet is released from the storage chamber and envelops the gelatin, causing its dissolution and consequent sensor release. (*F*) The sensor delivery robot crosses the obstacles to reach the target position, releasing the temperature sensor and retracting the carrier. (*G*) Design drawings of the liquid sampling robot and photos of the prototype. The liquid sampling robot consists of two parts: a sampling chamber sealed by ferrofluid and a chamber for storing the sampling chamber and ferrofluid droplet. (*H*) Schematic showing the liquid sampling robot collects liquid samples. (*I*) Snapshots of the sampling process of the liquid sampling robot in a solution of food dye. In all figures, the scale bar is 1 mm.

Magnetic solid-droplet systems also fulfill various functions, including microneedle patch actuation, sensor delivery, and liquid sampling. Microneedle patch is an alternative to oral and subcutaneous injections with unique advantages such as painless administration, good compliance, and fewer side effects (53). The microneedle patch and ferrofluid droplet are combined to construct a microneedle patch robot. Comprising four essential components, as depicted in Fig. 5*A*, this robot includes a bottom layer housing a ferrofluid chamber (diameter: 3 mm, height: 2 mm), a middle layer containing a chamber for low-boiling-point liquid storage (diameter: 3 mm, height: 1.5 mm), an upper layer featuring a sealing membrane for the low-boiling-point liquid, and a microneedle patch (diameter: 3 mm, height: 500 µm) (The fabrication process is shown in *SI Appendix*, Fig. S16*A*). The working principle of the microneedle patch robot is shown in Fig. 5*B*. Applying the RF field triggers the heating within the ferrofluid droplet, subsequently causing the low-boiling-point liquid (Novec 7000, Sigma-Aldrich Inc.) to vaporize. This vaporization exerts upward pressure on the sealing film and the upper layer of the microneedle patch. When the RF field is deactivated, the low-boiling-point liquid cools and condenses, causing a decrease in pressure inside the sealed chamber, after which the microneedle patch returns to its initial state. The experimental results in Fig. 5*C* and Movie S5 validate the design concept: At *t* = 0 s, the microneedle is in the initial position, as indicated by the red dashed line. Then, the RF field (650 A, 338 kHz) is activated, causing the microneedle patch to move upward by about 250 μm at *t* = 41 s. Afterward, upon deactivation of the RF field, the microneedle patch returns to the initial position at *t* = 62 s. Reactivating the RF field causes the microneedle patch to move upward by 250 μm again.

Sensing the physiological properties of soft biological tissues is paramount in gaining insights into tissue development, facilitating disease diagnosis, and advancing therapeutic interventions (54). Despite wireless miniature sensors hold promise for healthcare monitoring, they often lack mobility, and their deployment is typically invasive (55, 56). To overcome these challenges, our miniature solid-droplet systems can serve as delivery platforms for advanced biosensors, enabling precise deployment and long-term monitoring of physiological parameters. A miniature temperature sensor carrier is created by combining a microchip (a miniature RFID transponder with an integrated temperature biosensor) with a ferrofluid droplet. This robot consists of three key components: a bottom layer housing a ferrofluid chamber (diameter: 3 mm, height: 2 mm), a middle layer incorporating gelatin for bonding, and an upper layer containing the temperature sensor (diameter: 1.5 mm, length: 10 mm) (Fig. 5*D*, the fabrication process is shown in *SI Appendix*, Fig. S16*B*). The operational concept of the sensor carrier is illustrated in Fig. 5*E*. The ferrofluid droplet within the chamber contains enzymes capable of dissolving gelatin. When the carrier transports the temperature sensor to the targeted location, the ferrofluid droplet separates from the cavity and envelops the gelatin. The enzymes within the ferrofluid droplet then facilitate the release of the temperature sensor by dissolving the gelatin. Subsequently, the ferrofluid droplet returns to the cavity, allowing it to be driven back to its initial position. Fig. 5*F* demonstrates the functionality of the temperature sensor carrier as it navigates through obstacles to reach the designated yellow target area. After releasing the temperature sensor, the ferrofluid droplet together with the cavity returns to its initial position (Movie S6). Importantly, this design is versatile and not limited to temperature sensors. Similar approaches can be employed to deliver various other sensors, including those for measuring pH, biomarkers, and more. This sensor delivery platform offers a promising avenue for minimally invasive, long-term physiological monitoring deep inside human bodies.

Liquid biopsies have emerged as a groundbreaking advancement in clinical oncology over the last decade, gradually replacing invasive techniques for diagnosing and monitoring cancers (57, 58). Our proposed solid-droplet system can collect liquid samples and facilitate the development of liquid biopsy. The fluid sampling robot consists of two main parts: a large storage chamber that encases ferrofluid droplets and a sampling chamber for liquid biopsy (Fig. 5*G*, the fabrication process is shown in *SI Appendix*, Fig. S16*C*). The sampling principle is demonstrated in Fig. 5*H*. Initially, the sampling chamber is filled with a ferrofluid droplet (sealing droplet). Subsequently, another ferrofluid droplet (referred to as a driving droplet) used for propulsion enters the storage chamber. The driving droplet merges with the sealing droplet, transporting the sampling chamber to the targeted region containing the liquid to be sampled. The merged ferrofluid droplet then separates from the sampling chamber, allowing the sampling chamber to be filled with the local liquid. Afterward, the ferrofluid from the sampling chamber recombines with it, sealing the sampled liquid inside to prevent contamination. The sampling chamber is returned to the storage chamber for retraction. Fig. 5*I* and Movie S7 demonstrate the whole sampling and releasing process. The driving droplet deforms into the storage chamber at *t* = 0 s (*Top* row). At *t* = 93 s, the driving droplet transports the sampling chamber out and combines with the sealing droplet inside the sampling chamber to prevent other fluids from entering the chamber. Then, when the merged droplet carries the sampling cavity into the sampling area, it is driven to release its sampling cavity. At this time, the ferrofluid droplet inside the sampling cavity is also separated from it so the sampled liquid can enter the inside (*t* = 130 s). Then, the merged droplet combines with the sampling chamber again to seal the sampled fluid inside to prevent it from being contaminated (*t* = 158 s). The merged droplet then carries the sampling chamber back to the storage chamber. The *Bottom* row of Fig. 5*I* shows the process of releasing the sampled liquid from the sampling chamber: The yellow liquid spreads out when the ferrofluid separates from the sampling chamber.

### Modularity and Selective Control of Magnetic Solid-Droplet Systems.

**Fig. 6.**
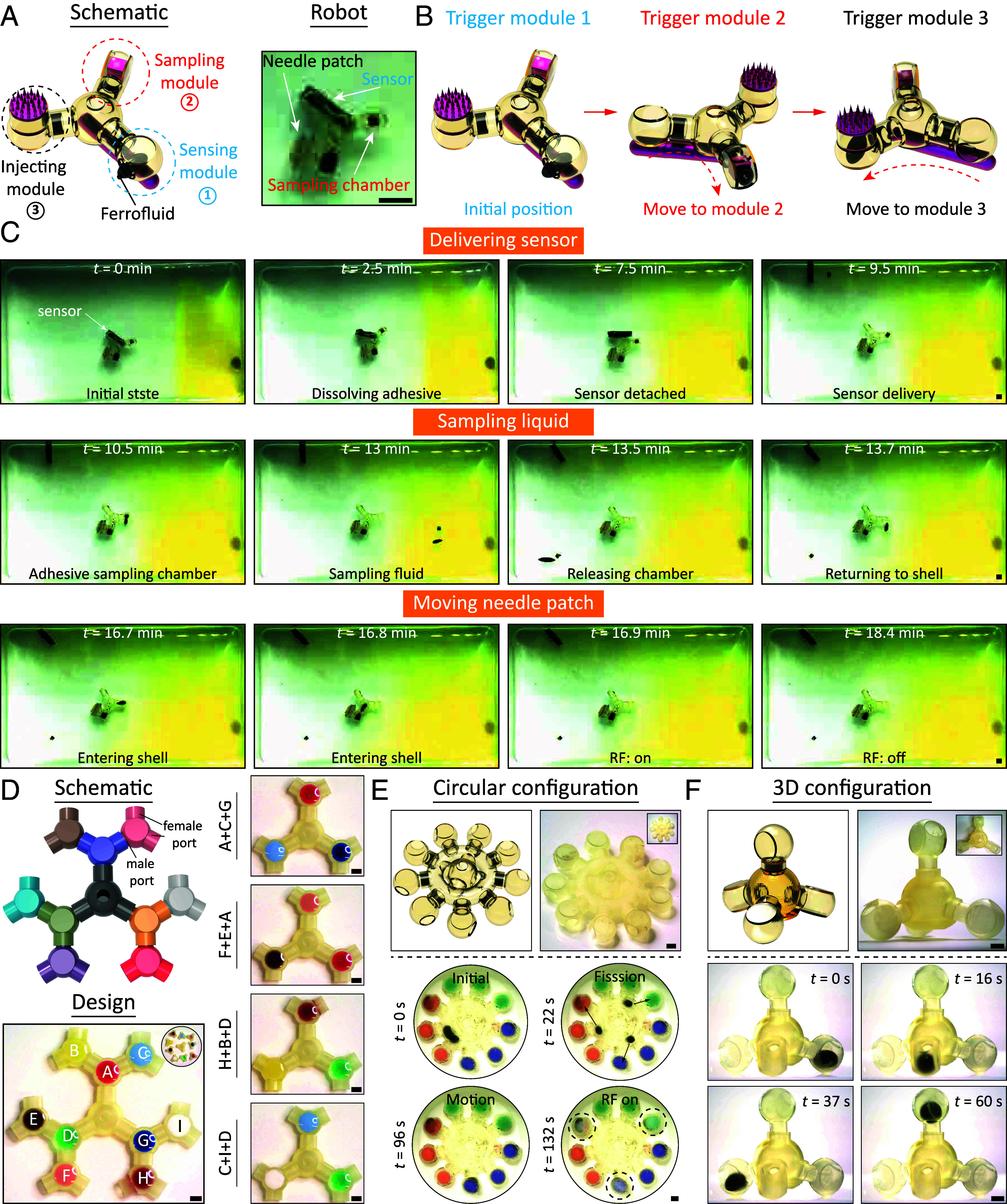
Demonstration of modularity and selective control of solid-droplet system. (*A*) Design drawings of the multifunctional solid-droplet system and photo of the prototype. The multifunctional solid-droplet system consists of a temperature sensor, a microneedle patch array, and a sampling chamber. (*B*) Principles of selective control for the multifunctional solid-droplet system. (*C*) Snapshots of the multifunctional solid-droplet system sequentially accomplish sensor delivery, liquid sampling, and microneedle patch array motion functions. (*D*) The schematic diagrams and experimental photos show that different modules, like building blocks like Legos, can be assembled on demand to create a multifunctional solid-droplet system. Each functional module follows a standard structure with one male port and two female ports, allowing them to be interconnected to achieve a wide range of functionalities. (*E*) Design drawings and photographs of a solid-droplet system with a circular configuration. Within the circular configuration, the ferrofluid droplet utilizes its splitting characteristics to trigger three color-changing regions simultaneously. (*F*) Design drawings and prototype photographs show that the solid-droplet system has a 3D configuration, and the ferrofluid droplet can traverse each functional cavity in three dimensions via a magnetic gradient. In all figures, the scale bar is 1 mm.

While some of the existing magnetic miniature robots can perform multiple functions (16, 17, 19, 20, 22, 28), they are often incapable of handling scenarios requiring the sequential or simultaneous execution of various functions in a complex task. For example, maneuvering the small device to a targeted location may involve sequentially diagnosing, treating, and sampling at the target. Nonetheless, the magnetic component is typically immobilized inside the body of conventional magnetic miniature robots. Consequently, each part of the robot containing magnetic components reacts collectively to the external magnetic field. This lack of independence among magnetic components in different parts makes integrating multiple functions into a magnetic robot challenging. One advantage of using ferrofluid droplets is that they can be combined with and separated from various functional modules, enabling the assembly of different functions in a solid-droplet system. Previous demonstrations have illustrated that the solid-droplet system can perform functions such as microneedle patch, sensor delivery, and liquid biopsy separately. In this context, we aim to integrate all three functions into a single solid-droplet system. As depicted in Fig. 6*A*, the schematic illustrates the assembly of modules with identical specifications to create a multifunctional solid-droplet system. A ferrofluid droplet, the magnetic component, can be moved sequentially from a sensor delivery module to a liquid sampling module and then to a microneedle patch module, independently triggering each functional module (Fig. 6*B*). Fig. 6*C* and Movie S8 show the sequential movement of the multifunctional solid-droplet system to various targeted locations where each of the three functions is performed. At *t* = 2.5 min, the ferrofluid droplet, containing dissolving enzymes, enters the inner cavity of the sensor delivery module, exits from it, and adheres to the sensor-cavity connection, eventually releasing the sensor due to dissolution. At *t* = 10.5 min, the ferrofluid droplet enters the sampling module, collects samples from the target area, and returns to the robot body. Finally, when *t* = 16.9 min, the ferrofluid droplet enters the inner cavity, propelling the robot to the injection site, where it initiates the microneedle patch under the RF field.

The modularity and selective control of solid-droplet systems hold promise for a wide range of applications, extending far beyond the scope of this study. With an array of functional modules available, we have the flexibility to combine them as needed to create the ideal solid-droplet system. Think of it as akin to assembling Lego blocks, where each functional module follows a standard design featuring one male and two female ports. As illustrated in Fig. 6*D*, we have a tree-like planar fractal system comprising nine dummy functional modules and one central module for ferrofluid droplet storage. These functional modules are labeled “A” through “I”, and we can combine them in various ways to meet specific requirements, for instance, “A+C+G” or “C+I+D” combinations. Beyond the dendritic configuration, we can explore a circular arrangement, as depicted in Fig. 6*E*. Here, nine functional modules and one central storage module are arranged in a circular planar layout. The modules are grouped into three sets, each distinguished by a different color: red, green, and blue, achieved using thermochromic pigments. Initially, the ferrofluid droplet is stored in the central region and can transport the assembly in a specific direction when subjected to an external magnetic field (*SI Appendix*, Fig. S17). The ferrofluid droplet can enter various modules marked with different colors and cause their pigment to fade sequentially when subjected to the RF field (*SI Appendix*, Fig. S18). Additionally, it can divide into three subdroplets, with each one entering color-marked modules, and the RF field causes their colors to fade simultaneously (Movie S9). The control strategy for inducing ferrofluid droplet splitting and selectively driving subdroplets is shown in *SI Appendix*, Fig. S19. As illustrated in the lower panel of Fig. 6*E*, at *t* = 0 s, the ferrofluid droplet is a single entity. Then, at *t* = 22 s, the magnetic field divides it into three subdroplets. Subsequently, the magnetic field guides these three subdroplets to enter the blue, red, and green temperature-sensitive modules sequentially. Under the influence of the RF field, all three modules change color to white simultaneously. Apart from the planar configuration, we have also developed 3D assembly designs. As depicted in Fig. 6*F*, this includes the assembly of a central storage cavity for ferrofluid droplets and three dummy functional modules into a three-pronged conical structure (Movie S9). The magnetic field can be adjusted to guide the ferrofluid droplet through the functional modules sequentially at various positions on the assembly. When subjected to an external magnetic field, the ferrofluid droplet can transport the assembly in a specific direction (*SI Appendix*, Fig. S20).

## Discussion

We have developed a generalized mechanism for loading and separating magnetic components in magnetic miniature robots. This generic mechanism is achieved using magnetic liquid materials (ferrofluid droplets) that can adhere to and then detach from shells of different materials. We further demonstrate that different functional solid-droplet systems can be created when varying the shell’s material, size, and structure and then utilizing the fluidity, fission/fusion, and magnetic response properties of ferrofluid droplets. Five exemplary applications demonstrate this mechanism’s feasibility in building magnetic robots: 1. Cell spheroid shell/scaffold delivery robot: By integrating ferrofluid droplets with submillimeter cell spheroid shells or scaffolds, we have engineered cell delivery robots capable of precisely and efficiently delivering these structures. Notably, the cell scaffolds here are made of nondegradable materials, which can be replaced with degradable or absorbable materials for in vivo cell delivery. 2. Stent delivery robot: Ferrofluid droplets seamlessly integrated with millimeter-scale stent structures facilitate the creation of stent delivery robots tailored for stent delivery purposes. 3. Microneedle patch robot: Combining ferrofluid droplets with a microneedle patch array results in the development of a microneedle patch robot adept at performing precise injections with accuracy and control. 4. Sensor delivery robot: We have engineered sensor delivery robots by integrating ferrofluid droplets with miniature temperature sensors. Importantly, our system can readily accommodate various miniature sensors for versatile sensing applications. 5. Liquid sampling robot: When synergized with a sampling structure, ferrofluid droplets empower the construction of solid-droplet systems optimized for liquid biopsy procedures. The ferrofluid’s ability to effectively seal the sampled liquid within a secure chamber mitigates the risk of contamination.

Furthermore, ferrofluid’s fluidity and fission-fusion properties endow the solid-droplet system with modularity and selective control. Modularity: Analogous to assembling modular building blocks, our solid-droplet systems are customizable. This modularity empowers users to tailor the robot’s functionality by seamlessly gathering diverse functional modules to meet specific needs, enhancing versatility and utility. Selective control: The ferrofluid droplet can independently access distinct functional module areas to initiate precise functions. Additionally, its unique capability to divide into multiple subdroplets enables concurrent activation of various functions within different functional modules, adding a heightened level of sophistication to our robots’ operational capabilities. Importantly, magnetic soft robotic swarms cannot match the capabilities of ferrofluid droplets in our system, as they struggle in triggering multiple functions at the same time and face issues like aggregation (*SI Appendix*, Figs. S21 and S22), despite their potential for quasi-fluidity. In conclusion, our research introduces a construction strategy for magnetic miniature robots, leveraging ferrofluids to redefine versatile, modular, and decoupled solid-droplet systems for various applications.

The proposed mechanism for loading and separating magnetic components is a versatile technology that can be seamlessly integrated with a wide range of shells, spanning from submillimeter (0.8 mm) to centimeter scales (1.5 cm) and comprising diverse materials, from biomaterials to polymers. These shells can take on various forms, including planar and 3D structures, allowing for the creation of solid-droplet systems with adaptability. Solid-droplet systems, constructed based on the separable mechanism between the magnetic component and the structural body, offer several advantages over existing magnetic miniature robots: 1. Enhanced biocompatibility: The separability of the functional body from the magnetic component reduces biocompatibility challenges, especially for miniature robots that require prolonged placement. The magnetic fluid can be detached and recycled if the robot body is biocompatible or bioabsorbable. 2. Modular assembly: Solid-droplet systems can be assembled like Lego blocks, enabling various functional combinations and reconfigurations tailored to specific requirements. 3. Decouple: These robots can achieve selective control by independently controlling different functional areas or even enabling concurrent operation of multiple functional areas.

However, developing the next generation of solid-droplet systems presents some notable challenges: 1. Complex deformation: Current solid-droplet systems lack the capability for complex deformation typically seen in traditional soft-bodied robots. The utilization of temperature-sensitive materials holds promise for achieving such complex deformations. 2. Exploiting other properties of ferrofluid: Presently, solid-droplet systems rely primarily on the ferrohydrodynamic instabilities and magnetic heating effect of ferrofluids. Future advancements should explore other unique properties of ferrofluids to create robots with additional functionalities. 3. Exploration of alternative magnetic materials: Current solid-droplet systems use oil-based ferrofluids as magnetic components; future investigations may consider alternative magnetic materials like magnetorheological fluids or magnetic slime materials. Furthermore, our solid-droplet systems can seamlessly integrate with traditional soft robots, leveraging their inherent advantages. This approach significantly expands the design possibilities and enhances device functionality. It opens opportunities for creating highly functional devices with intricate designs explicitly tailored to meet the demands of various biomedical applications.

## Materials and Methods

### Materials.

Oil-based ferrofluids with a density of 1.43 g/cm^3^ and dynamic viscosities of 8 mPa s were procured from Ferrotec Corporation, with detailed information provided in *SI Appendix*, Table S1 (the magnetic hysteresis curve is shown in *SI Appendix*, Fig. S23). Ecoflex 00-30, Sylgard 184, Photoreactive resin Clear V4, Photoreactive resin PEGDA, and Dopamine hydrochloride were obtained from Smooth-on Inc., Dow Chemical Company Ltd., BMF Precision in China, and Aladdin Chemical Co. Ltd., respectively. Sandpapers of varying roughness levels were acquired from Conrad. Dulbecco’s modified Eagle’s medium, fetal bovine serum, 0.25% trypsin-EDTA, DAPI, phosphate-buffered saline, and Nunclon Sphera 96-well U-shaped-bottom microplates were purchased from Gibco, a division of Thermo Fisher Scientific. The Make to Stock (MTS) assay (ab197010) was sourced from Abcam. All chemical components were used without the need for further purification.

### Magnetic Actuation Setup.

As depicted in Fig. 2*B*, the magnetic actuation setup consists of a 7-degree-of-freedom robotic arm (Panda Research, Franka Emika GmbH), a stepper motor (535-0372, RS Components GmbH), and a 50 mm cubic NdFeB permanent magnet (maximum magnetic field strength can reach 700 mT). The communication infrastructure was established using the Robot Operating System. This magnetic actuation system can achieve 3D magnetic actuation in space.

### Contact Angle, Adhesion, and Shear Force Measurements.

The contact angle measurement shown in Fig. 3*A* was carried out using a commercial contact angle measurement setup (Drop Shape Analyzer DSA, Krüss GmbH) in the sessile drop mode. Samples, including agar, glass, resin, PMMA, and metal, were prepared as 5 mm by 5 mm patches. For each test trial, the contact angle was determined at least five times with a fixed ferrofluid droplet volume of 5 µL, and the average of these measurements was taken as the contact angle.

Adhesion and shear force tests were performed using a custom-designed experimental setup, as depicted in *SI Appendix*, Fig. S2*A*. These tests involved the use of two load cells (GSO-25, Transducer Techniques LLC; and LSB200, FUTEK Inc.) connected to a probe, oriented vertically and horizontally, for measuring adhesion and shear forces, respectively. These load cells were attached to a linear motor stage capable of vertical motion. The specimen under examination was positioned on a glass slide mounted on a linear motor, functioning as a tangential translation stage.

The adhesion test consisted of two steps: loading and retracting, both exclusively involving vertical probe motion. In contrast, the shear force test included three steps: loading, translation, and retracting. The translation phase involved the tangential movement of the specimen stage, as shown in *SI Appendix*, Fig. S2*B*. For the experimental tests, a 30 µL droplet of ferrofluid was confined at the base of the probe using a 2 mm diameter permanent magnet to ensure alignment with the substrate. Five distinct tests were conducted on agar, glass, resin, PMMA, and metal substrates, respectively.

In the adhesion test, the approaching and retracting speeds of the probe were set to 1 μm/s, with a preload of 3 mN. For the shear force test, the approaching and retracting speeds of the probe were also set to 1 μm/s, with a preload of 3 mN and a contact time of 5 s. The tangential displacement and speed were 0.4 mm and 1 μm/s, respectively.

### Modeling the Dynamics of Ferrofluid.

The shape of a ferrofluid droplet is governed by a combination of magnetic, gravitational, and interfacial tension forces, with the assumption that forces related to wetting are negligible (59). The interfacial free energy is minimized when the droplet adopts a spherical shape. However, gravitational, and magnetic forces act to alter this ideal shape. The magnetization of the ferrofluid induces elongation of the droplet in the direction of the magnetic field, and the relative influence of this effect compared to interfacial tension can be quantified using a dimensionless parameter denoted as *S*:

[1] $$S=\mu_{0}M^{2}V^{\frac{1}{3}}\sigma^{-1}.$$

Here, *μ*_0_ represents vacuum permeability, *M* is the magnetization of the ferrofluid droplet, *V* is the volume of the droplet, and *σ* is the interfacial tension between the ferrofluid and the surrounding fluid. We positioned a permanent magnet beneath the ferrofluid droplet, which generated a nonuniform magnetic field that exerted a vertical magnetic force density, denoted as $f_{M}$:

[2] $$f_{M}=\mu_{0}(\bar{M}\cdot \nabla )\bar{H}=\mu_{0}MdH/dz.$$

Here, *H* signifies the external magnetic field. We approximate *M* and the vertical field gradient, d*H*/d*z*, as constant across the volume of the droplet, calculated at its center. This magnetic force combines with the gravitational force density, $f_{G}$:

[3] $$f_{G}=\Delta \rho g.$$

Here, Δ*ρ* represents the density contrast between the ferrofluid and the surrounding fluid, while *g* stands for the gravitational acceleration. The resultant normal force density, $f_{N}$, which is the sum of $f_{G}$ and $f_{M}$, acts to compress the droplet against the substrate, causing it to flatten. This flattening effect can be quantified concerning interfacial tension using the effective Bond number, denoted as $B_{e}$:

[4] $$B_{e}=f_{N}V^{\frac{2}{3}}\sigma^{-1}.$$

The interaction between interfacial, gravitational, and magnetic forces gives rise to intriguing phenomena, including field-induced instabilities. A classic example is the Rosensweig instability, where a uniform vertical magnetic field leads to the formation of a macroscopic array of spikes on the surface of a horizontal ferrofluid. The critical wavelength determines the periodicity of this spike array, $\lambda_{c}^{\text{Rosensweig}}=2\pi \sqrt{\sigma /f_{G}}$. In the case of a nonuniform magnetic field generated by a permanent magnet, the critical wavelength can be similarly expressed as $\lambda_{c}=2\pi \sqrt{\frac{\sigma }{f_{N}}}$.

### Thermal Characterization of the Ferrofluid.

The temperature of the ferrofluid droplet under different environments was measured when actuated by using an infrared camera (ETS320, Teledyne FLIR) (*SI Appendix*, Fig. S12*A*). The ferrofluid droplet was first positioned visually at the center of the RF coil at the appropriate height. As the camera has a fixed focus distance of 7 cm, the infrared camera was placed 7 cm above the ferrofluid droplet during the experiments. Before the tests, the camera underwent a calibration process using reference points: melting ice and boiling water. Subsequently, 10 μL of ferrofluid was carefully introduced into the same container, in both empty and water-filled conditions. The temperature variations of the ferrofluid droplets, subjected to a continuous input of 338 kHz, were then recorded, and analyzed.

## Supplementary Material

Appendix 01 (PDF)

Movie S1. Mechanisms for assembling, actuating, and separating ferrofluids with solid shells of different sizes.

Movie S2. Selective assembly of ferrofluid droplets with different solid shells.

Movie S3. Magnetic sub-millimeter scale solid-droplet system for cell transplantation.

Movie S4. Magnetic millimeter scale solid-droplet system for stent delivery.

Movie S5. Magnetic millimeter scale solid-droplet system serves as microneedles patch.

Movie S6. Magnetic millimeter scale solid-droplet system serves as sensor delivery robot.

Movie S7. Magnetic millimeter scale solid-droplet system serves as liquid sampling robot.

Movie S8. Multifunctional magnetic solid-droplet system with decoupling capability.

Movie S9. Magnetic solid-droplet system with different configurations.

## Data Availability

All data are provided in the manuscript and supporting information.
