# Supplementary material for: Versatile, modular, and customizable magnetic solid-droplet systems
Source: Proc Natl Acad Sci U S A. 2024 Aug 1;121(32):e2405095121. doi: 10.1073/pnas.2405095121 (PMC11317579; doi:10.1073/pnas.2405095121)
Supplement: Supplementary file 1 — Appendix 01 (PDF) [file pnas.2405095121.sapp.pdf]

Supplementary Information for

Versatile, modular, and customizable magnetic solid-droplet systems

Mengmeng Sun[1,#], Yingdan Wu[2,# *], Jianhua Zhang[1], Hongchuan Zhang[1], Zemin Liu[1,3], Mingtong Li[1], Chunxiang Wang[1,3], Metin Sitti[1,3 *]

[1] Physical Intelligence Department, Max Planck Institute for Intelligent Systems, 70569 Stuttgart, Germany
[2] State Key Laboratory of Robotics and Systems, Harbin Institute of Technology, Harbin 150001, China.
[3] School of Medicine and College of Engineering, Koç University, 34450 Istanbul, Turkey

[#] Equally contributing co-first authors
[*] Correspondence to: yingdanwu@hit.edu.cn; sitti@is.mpg.de

The PDF file includes:

Figs. S1 to S23
Table S1

Other Supplementary Material for this manuscript includes the following:
Movies S1 to S9

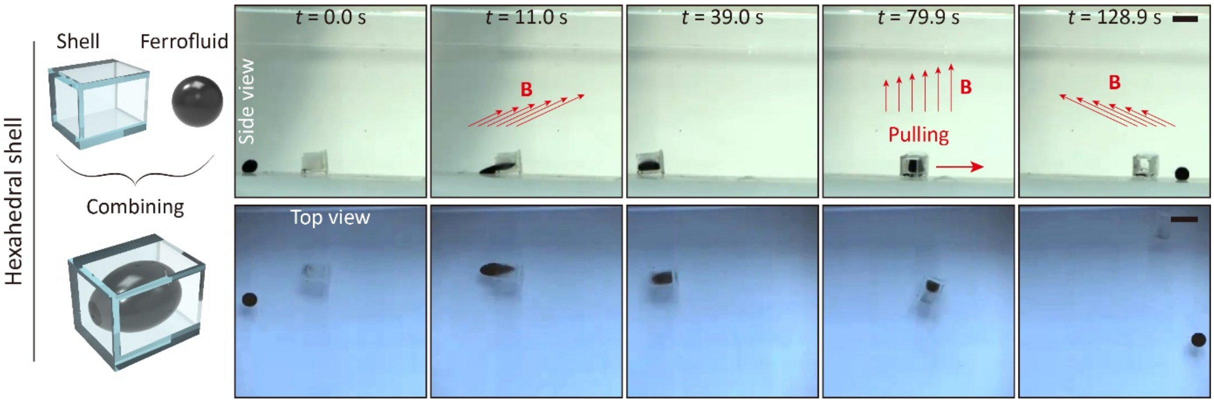

Fig. S1. The ferrofluid droplet and solid shell can be actively assembled. Solid shells (resin materials) with inlet dimensions greater than 1 mm can be combined and separated from the shell using active motion. For different shell shapes, the motion mode of the solid-droplet robot formed by the combination is different; for the square structure (below), dragging can be carried out by the magnetic field gradient force. Scale bars, 3 mm. Fabrication of helical, hexahedral, and spherical shells: initially, the structures were conceptualized and designed using computer-aided design software (SolidWorks, SolidWorks Corp. Inc., USA). The subsequent step involved crafting the resin shells with a 3D printer (NanoArch S130, BMF Precision, China), with the resin used being poly(ethylene glycol) diacrylate (PEGDA). To ensure the structural integrity of the shells and prevent any residual material from blocking the inlets, the resin shells underwent a two-minute ultrasound treatment involving polyethylene glycol. Following this, they were exposed to UV light for ten minutes. A hydrophilic treatment was then applied to enhance the shells' suitability for their intended applications.

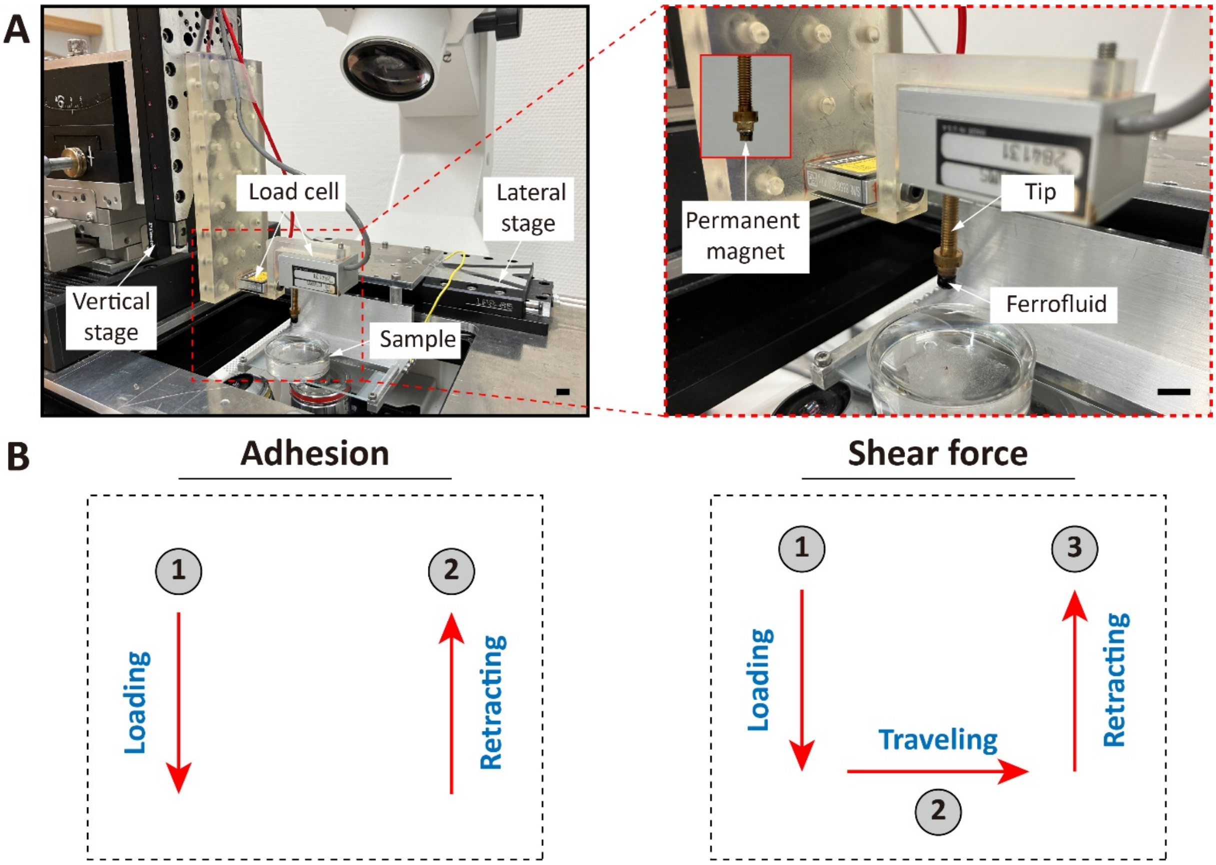

Fig. S2. Customized experimental setup for the adhesion and shear force measurement. **A.** Image of the experimental setup. The adhesion and shear force were measured using two high-precision load cells (GSO-25, Transducer Techniques, LLC) mounted on motorized vertical and lateral stages. The ferrofluid was bonded to the permanent magnet with a diameter of 2 mm connected to the load cell, and the substrate sample was placed on a microscope slide mounted on the lateral stage. **B.** Illustration of the process of the adhesion and shear force tests. Procedures of the adhesion measurement, including load, rest, and retract. The shear force measurement procedures include load, travel, and retract. Scale bars, 3 mm.

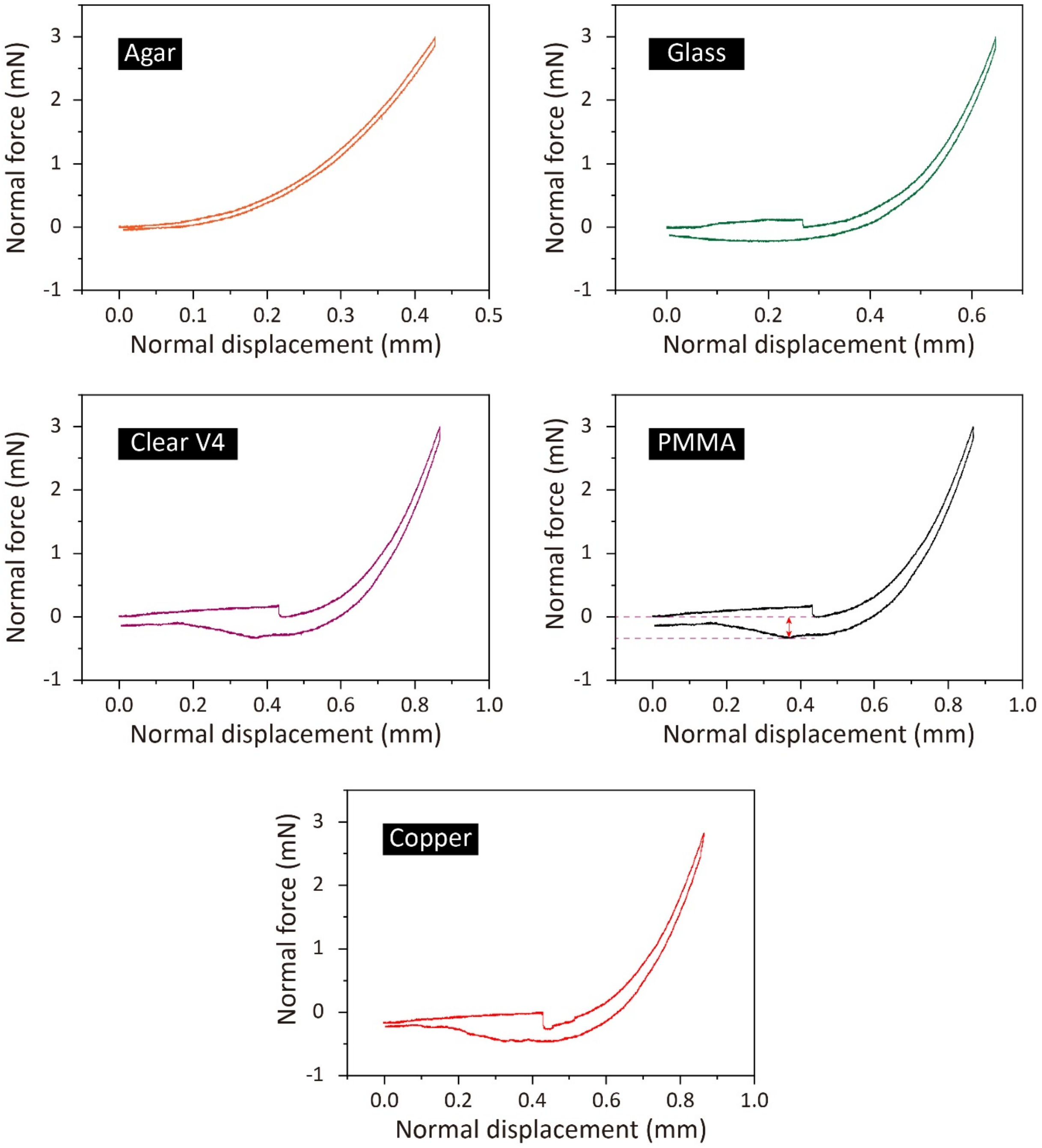

Fig. S3. Characterization of the adhesion of ferrofluid on different material substrates. Normal force curve measured for ferrofluid on different substrates under a preload of 3.0 mN and a contact time of 5 seconds. The adhesion is quantified using the maximum pull-off force (opposing normal force) denoted in the plot. The adhesion on the agar surface is around 0.05 mN, the adhesion on the glass surface is around 0.19 mN, the adhesion on the resin surface is around 0.23 mN, the adhesion on the PMMA surface is around 0.30 mN, and the adhesion on the metal surface is around 0.4 mN.

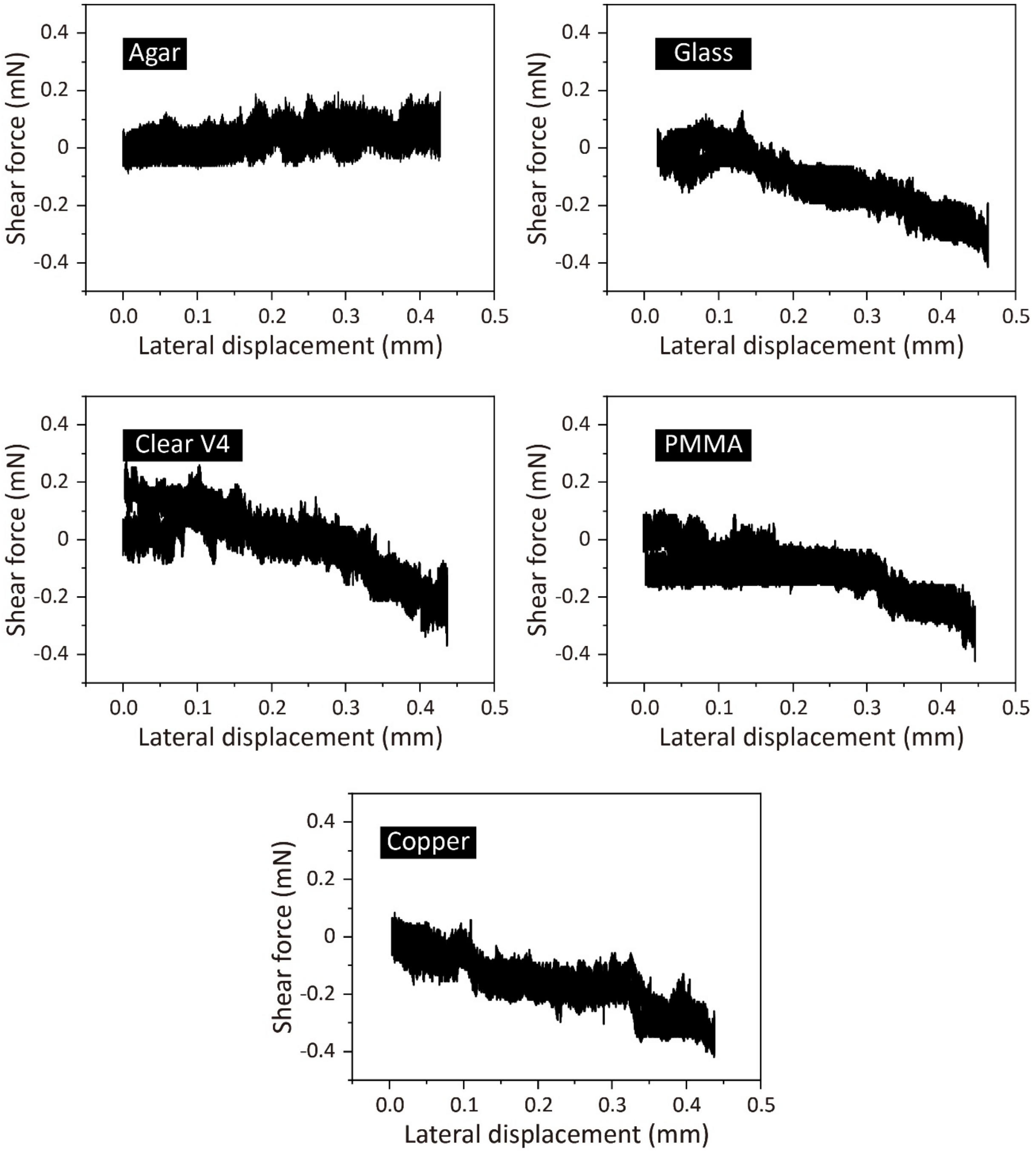

Fig. S4. Characterization of the shear force of ferrofluid on different material substrates. For the shear force test, the approaching and retracting speeds of the probe were set to 1 μm/s, while the preload and contact time were 3 mN and 5 s, respectively. The tangential displacement and speed were 0.4 mm and 1 μm/s, respectively. The shear force is quantified using the average of the forces measured on 0.4 mm. The shear force on the agar surface is around 0.01 mN, the shear force on the glass surface is around 0.20 mN, the shear force on the resin surface is around 0.15 mN, the shear force on the PMMA surface is around 0.22 mN, and the shear force on the metal surface is around 0.35 mN.

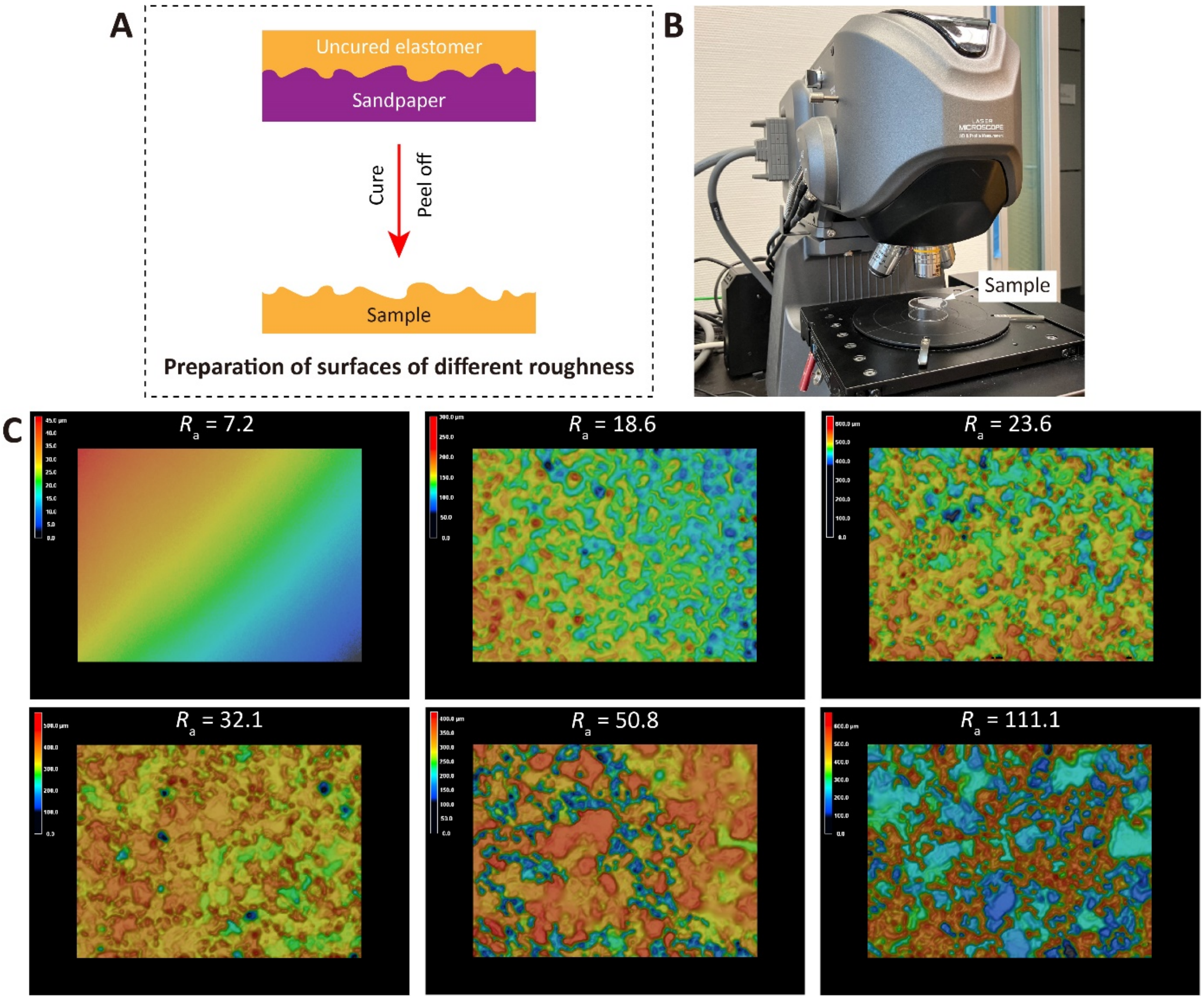

Fig. S5. Preparation and characterization of surfaces with different roughness. A. Schematic diagram of the preparation process of surfaces with different roughness. Uncured silicone was uniformly applied to the surface of sandpaper and peeled off after it was completely cured. **B.** Image of the characterization equipment (3D laser scanning confocal microscope, VK-X260K, KEYENCE). **C.** Morphological characterization of different roughness surfaces. Preparation and characterization of surfaces with different roughness: sandpaper with grits of 100, 150, 180, 240, 400, and 1000 was prepared and cut into 2 cm by 2 cm squares. Subsequently, a uniform layer of PDMS elastomer was applied to the surface of the sandpaper, and the assembly was placed in a drying oven at 90 °C for two hours. After the PDMS had fully cured, it was carefully peeled off from the sandpaper surface. Optical images and surface roughness data were acquired using a 3D laser scanning confocal microscope (VK-X260K, KEYENCE) with sub-nanometer resolution in the vertical direction and approximately 0.13 μm resolution in the lateral direction.

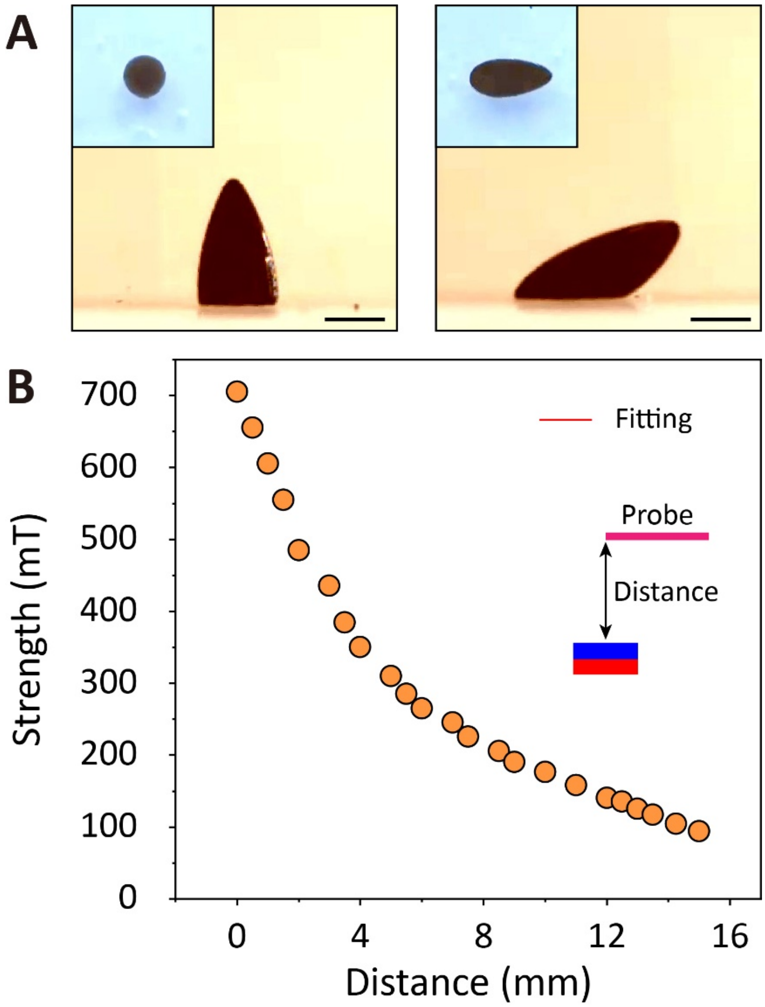

Fig. S6. The deformability of ferrofluid under various magnetic fields. A. Magnetic field-induced deformation of a ferrofluid droplet in immiscible deionized water. The top and side views of the ferrofluid droplet show that the magnet's different positions make its deformation morphologically different. Permanent magnet (diameter = 40 mm, height = 20 mm). **B.** The relationship between the strength of the magnetic field at the center of a permanent magnet in the vertical direction and the distance. Scale bars, 1 mm.

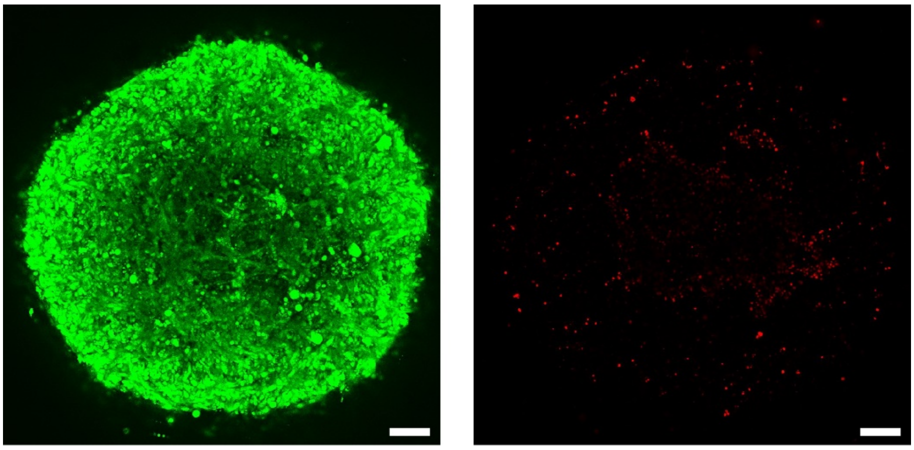

Fig. S7. Overlay image shows the viability of cell spheroid shell (green, live cells; red, dead cells). Scale bars, 100 μm. For the creation of cell spheroid shells, C2C12 cells (American Type Culture Collection-ATCC) were cultivated in a humidified environment at 37 °C with 5% CO_2. They were grown in 75-cm2 polystyrene cell culture flasks using MSC basal medium from ATCC, supplemented with the MSC growth kit for low serum (ATCC), 2% fetal bovine serum (Gibco), penicillin (25 IU ml−1), and streptomycin (25 μg ml−1). The adherent C2C12 cells were initially cultured until they reached 80 to 90% confluence, and then they were detached by treating them with a 0.25% trypsin-EDTA solution for 50 seconds at 37 °C. The trypsinization process was halted by adding twice the volume of DMEM supplemented with 10% FBS. The cell suspension was subsequently centrifuged at 1000 rpm for 5 minutes, and the resulting cell pellet was resuspended in 1 ml of DMEM containing 10% FBS. The cell density was determined using a hemocytometer. A C2C12 cell stock with a cell density of 5 × 106 cells/ml was prepared. Following this, 100 μl of the C2C12 stock solution was added to a non-adhesive 96-well plate. The 96-well plate was then incubated at 37 °C in a 5% CO_2 incubator for a minimum of 4 days to facilitate the generation of cell spheroid shells. Cell viability: To assess the viability of both the cell spheroid and the scaffold, a LIVE/DEAD viability/cytotoxicity kit (product no. R37601, Thermo Fisher Scientific, USA) was employed to stain live and dead cells, following the manufacturer's fluorescence microscopy protocol. In brief, a vial of live green dye was thawed and mixed with the dead red component to create a 2× working solution. This working solution was then added to the well containing the anchoring device, along with an equal volume of growth medium. The device and cells were incubated in this solution for 30 minutes at room temperature before imaging. A spinning disk confocal microscope (Nikon Instruments Inc., Eclipse Ti-E) was used to capture images of the live and dead cells.

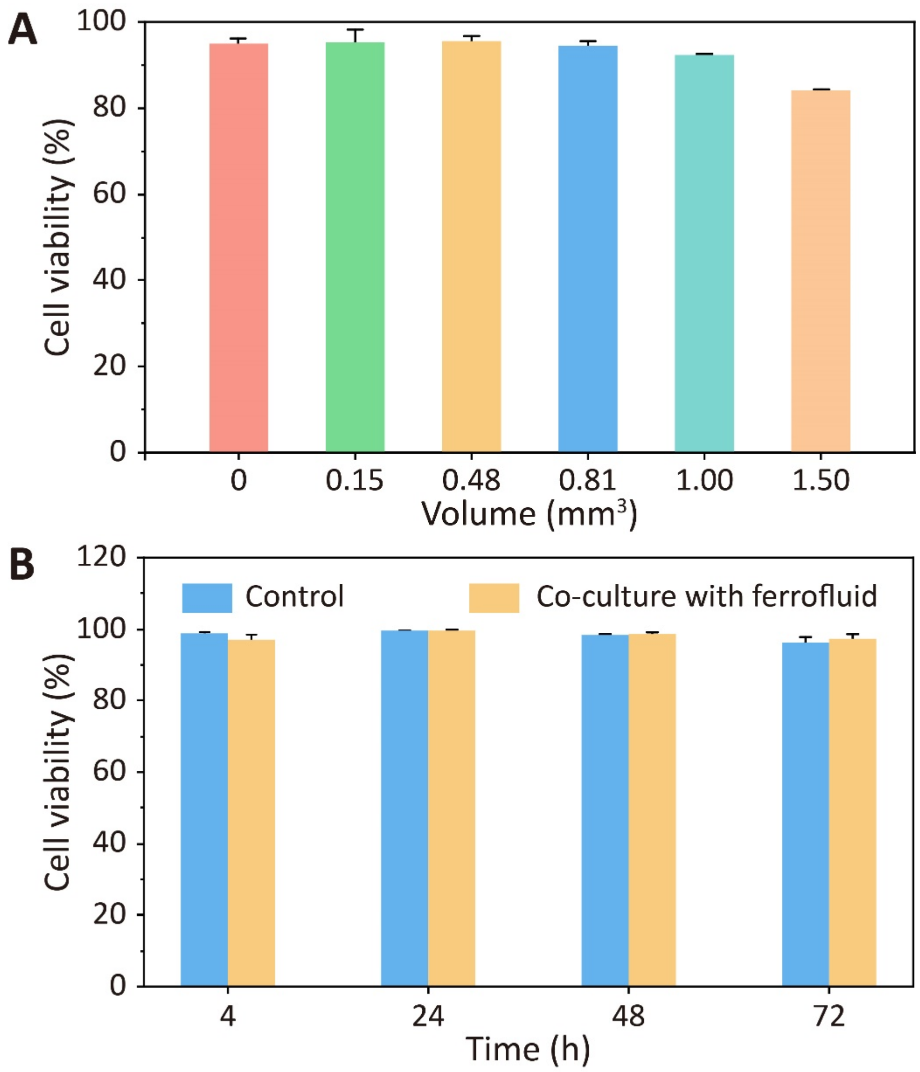

Fig. S8. Cell viability characterization. A. Cell viability of cell spheroid shells with different ferrofluid volumes after one hour of manipulation. **B.** Cell viability of cell spheroid shells with the same ferrofluid droplet volume (0.48 mm^3) after different manipulation times. Firstly, viability tests were performed on cell spheroid shells cultured with different volumes of ferrofluid for the same time 1 h. For a cell spheroid shell with a diameter of 0.9 mm. When the volume of ferrofluid is varied from 0 to 1.0 mm^3, it has almost no effect on the cell viability; when the volume of ferrofluid is 1.5 mm^3, it will inhibit the cell viability (Fig. S8A). Generally, 0.9 mm cell spherical shells, assembled droplets are generally used with a size of 0.5 mm^3. Secondly, viability tests were performed on cells cultured with the same volumes (0.5 mm^3) of ferrofluid for different time (from 4 h to 72 h). Figure S8B shows that a volume of 0.5 mm^3 of ferrofluid was incubated with the cell spherical shells for 72 h, and the viability of the shells was not affected. These results show that ferrofluid droplets do not affect cell viability during cell delivery.

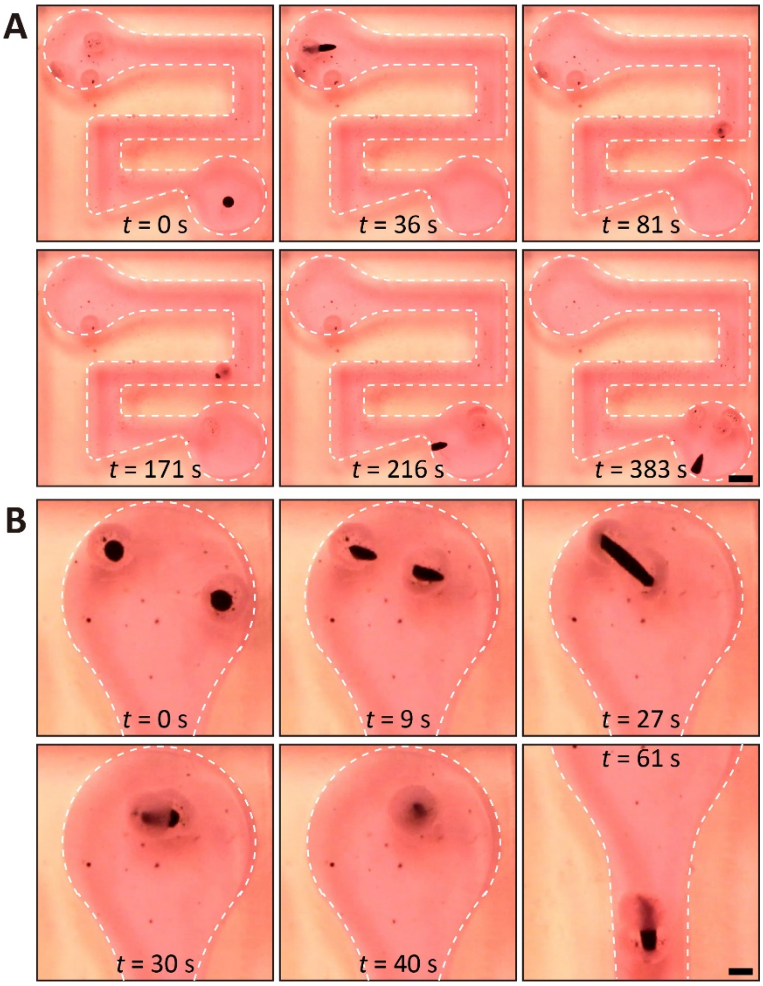

Fig. S9. Ferrofluid droplets transport cell spheroid shells. A. The ferrofluid transports two cell spheroid shells sequentially to a target location. **B.** The ferrofluid can carry two spheroid shells at the same time and assemble them into a ball for movement. Scale bars, 1 mm.

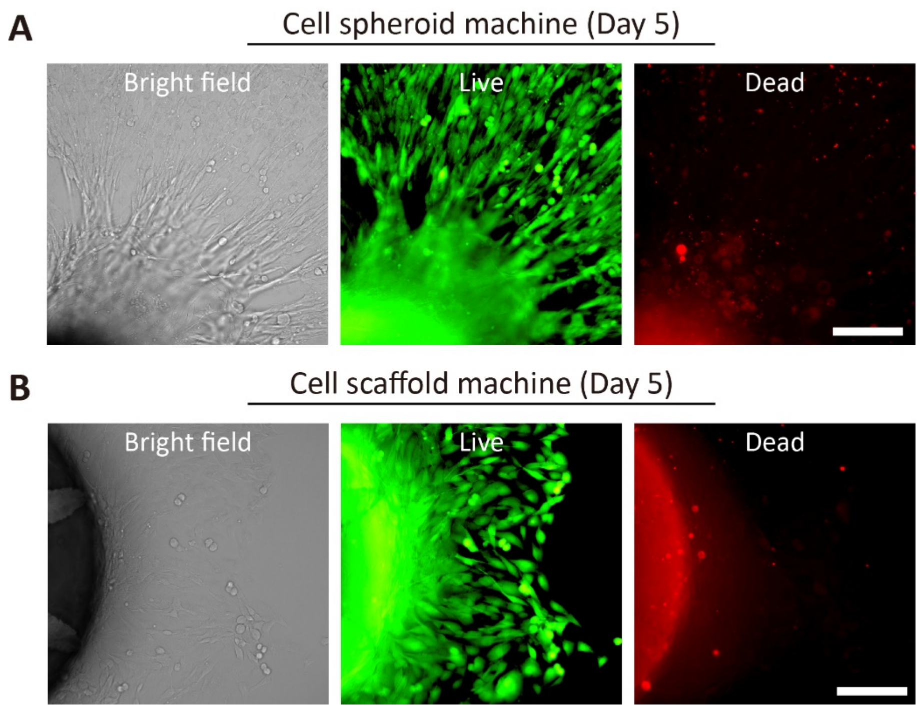

Fig. S10. Viability of released cell spheroid shell (A) and cell scaffold (B) after 5 days (green, live cells; red, dead cells). Scale bars, 100 μm. Cell scaffolds were manufactured using a 3D printer (NanoArch S130, BMF Precision, China) and poly(ethylene glycol) diacrylate (PEGDA) resin. Before cell seeding, the entire scaffold was immersed in a 1× phosphate-buffered saline (PBS) solution and sterilized under UV light for 4 hours. After sterilization, the scaffold was rinsed with PBS, and cells in suspension were seeded from the top in 50-μl volumes with a cell density of 5 × 106 cells/ml. The device with the entrapped cells was then transferred to a humidified environment at 37 °C with 5% CO_2 for a minimum of 3 days.

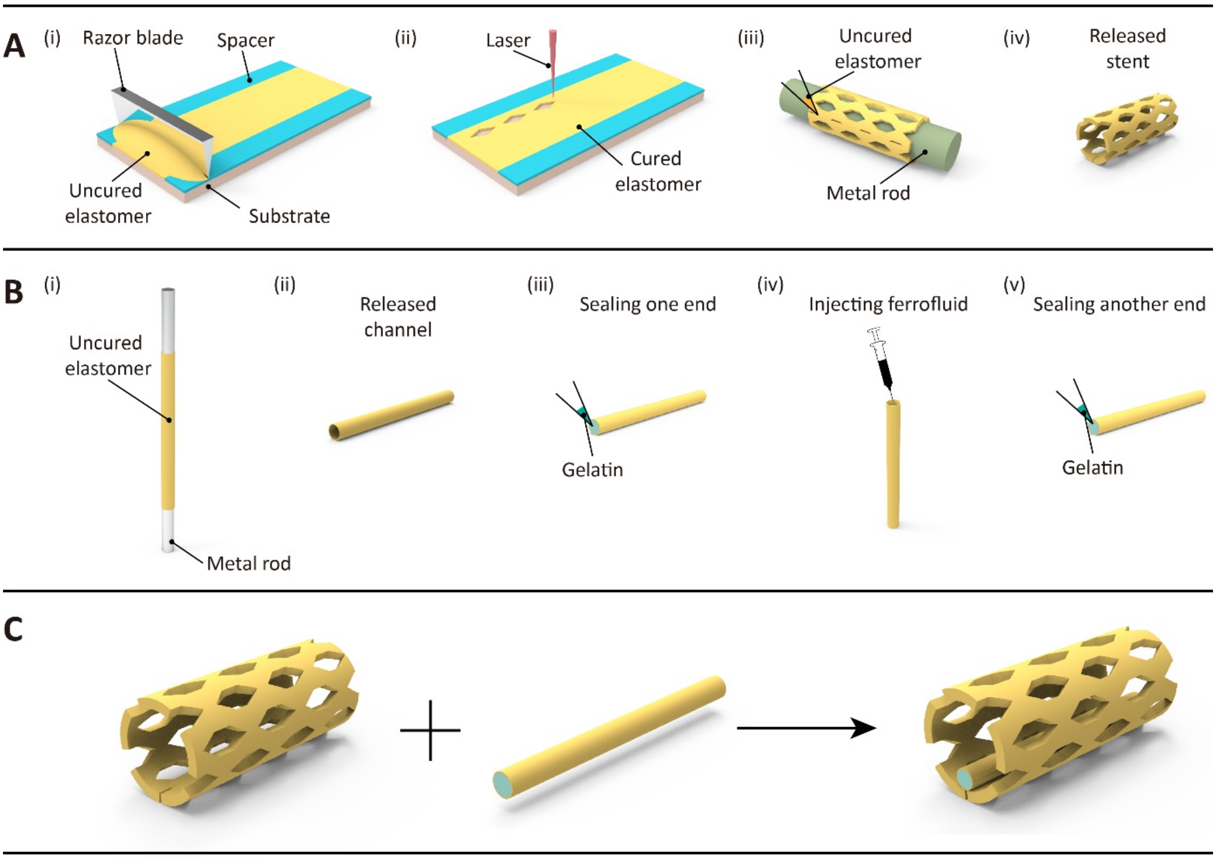

Fig. S11. Schematics of the fabrication process of the stent robot body. A. Illustration of fabricating the external stent-shaped body with a desired thickness. The Ecoflex-0030 elastomer was poured onto a PMMA substrate. A razor blade was used to scratch against the spacer for a uniform thickness. Then, the elastomer sheet was cut into a design shape with specified dimensions using a laser machine (LPKF ProtoLaser U3, LPKF Laser & Electronics AG). Finally, the flat sheet was crimped onto a metal rod and bonded with uncured silicone. **B.** Illustration of fabricating the interior channel for storing ferrofluid. Ecoflex-0030 elastomer was applied to the metal rod, then cured and released down the channel. One end of the channel was then sealed with gelatin, followed by injection of ferrofluid into the interior, and finally, the other end was also sealed with gelatin. **C.** Schematic showing the combination of a channel and an external stent shell to build a stent robot.

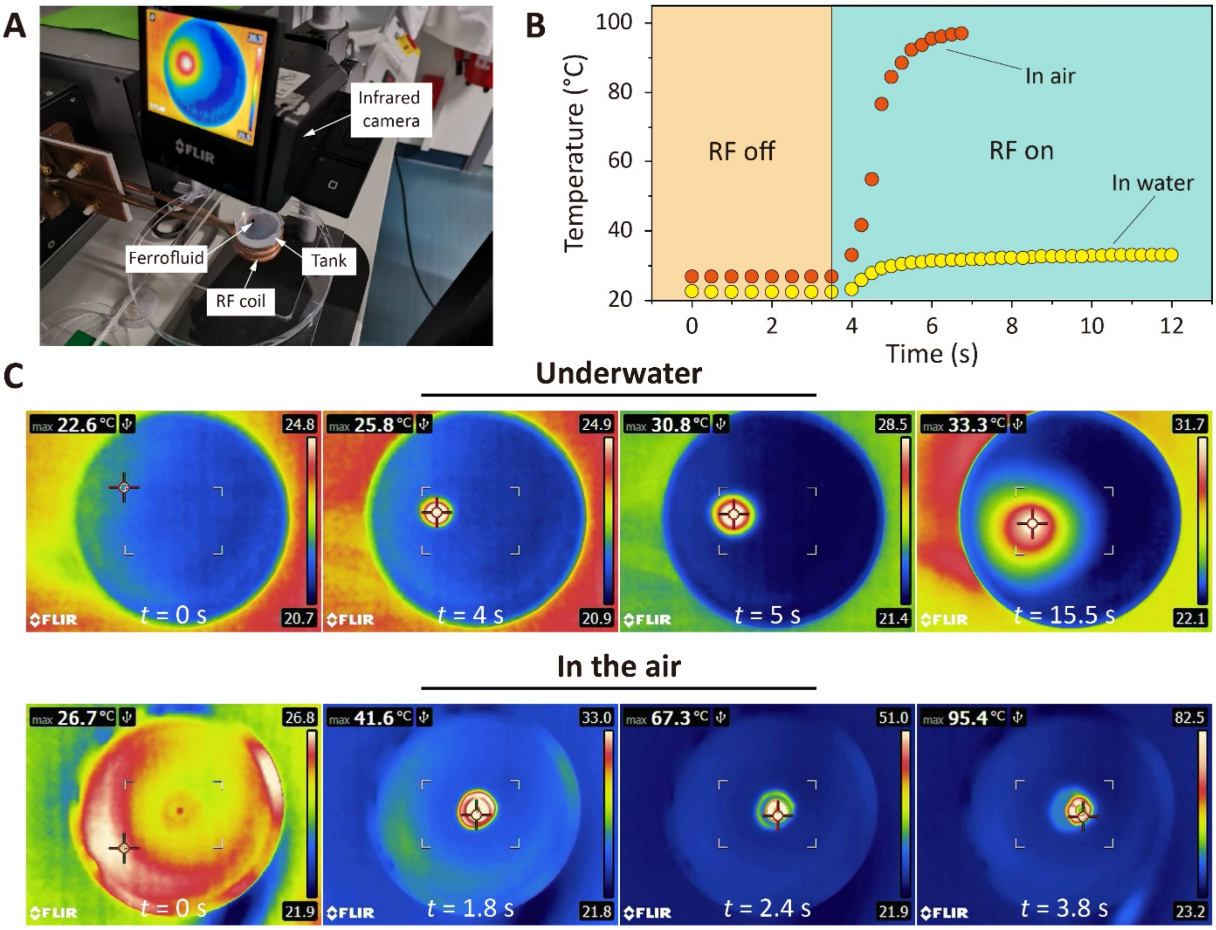

Fig. S12. Characterization of the thermal response of ferrofluids inside different environments. A. Experimental setup for characterization includes RF heating equipment (EASYHEAT, Ambrell Corporation), an infrared camera (Blackfly S USB3, FLIR Systems). **B.** An infrared imaging device observes the temperature of a ferrofluid droplet with time under an RF field of 750 kHz. **C.** Infrared camera images of the heating process underwater and air. The location of the hottest region, which corresponds to where the copper plate is located.

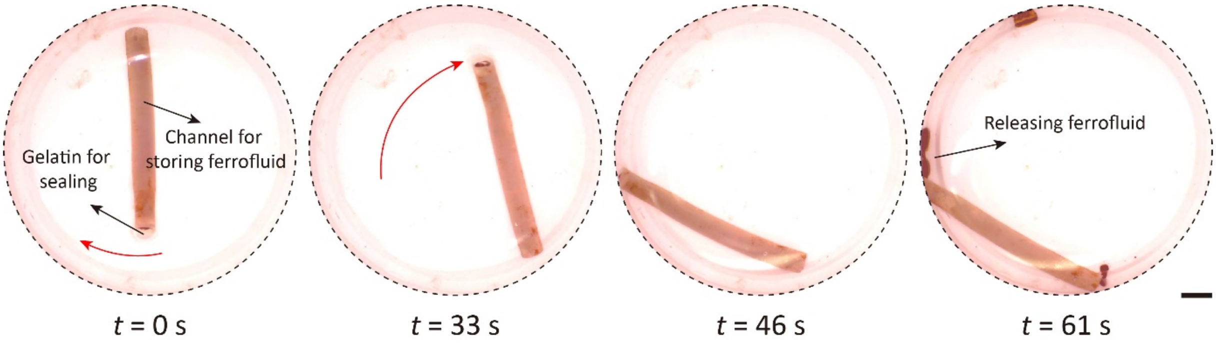

Fig. S13. Snapshot showing channels sealed at both ends with gelatin to store the ferrofluid, which will not leak the fluid when driven by a magnetic field. The stent robot dissolves the sealant at both ends and releases the stored ferrofluid under the RF fields. Scale bar, 1 mm.

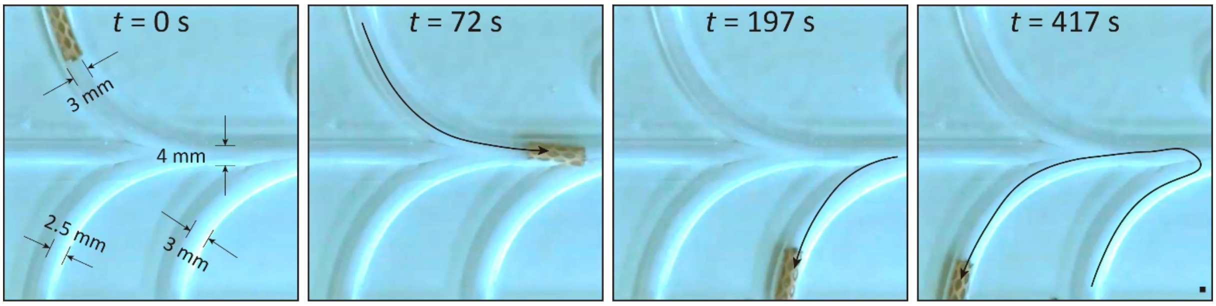

Fig. S14. Locomotion of stent robot in a tortuous route. The radius of the channel range is from 2.5 mm to 4 mm. Scale bars, 1 mm.

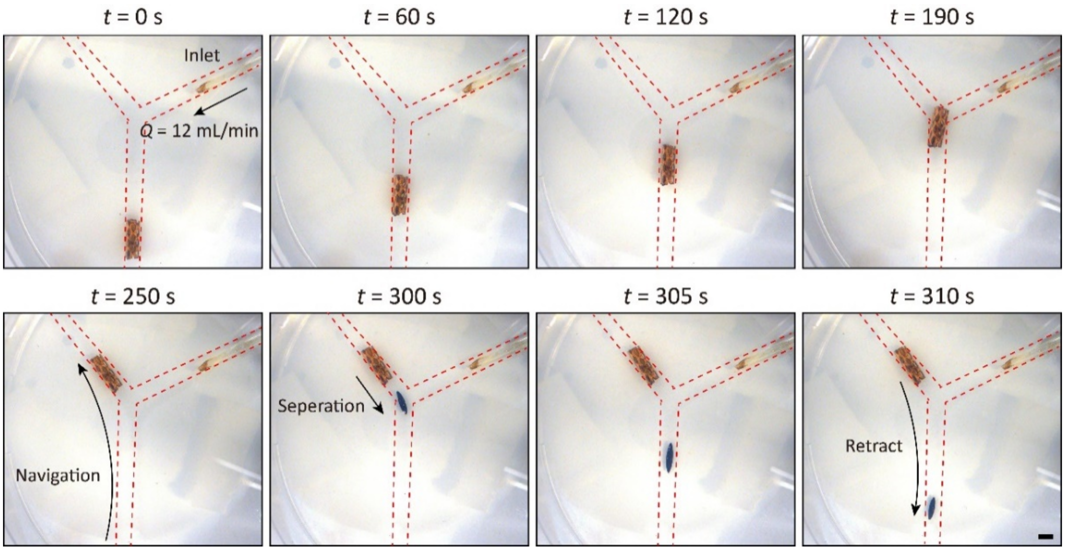

Fig. S15. Stent could be released in the tortuous channels under continuous flow. Scale bar, 4 mm. We test the ability of the stent robot to move in a fluid environment. We apply fluid with a flow rate of 12 mL/min to the bifurcating branches. After 250 s the stent robot moves to the target position and then turns on the RF heater. At t = 300 s, the ferrofluid droplets could be separated from the stent robot. At t = 310 s, the droplets are finally retracted. Preliminary experiments prove that our proposed method is feasible in a fluidic environment, but its application in a real blood flow environment will be further verified in our next work.

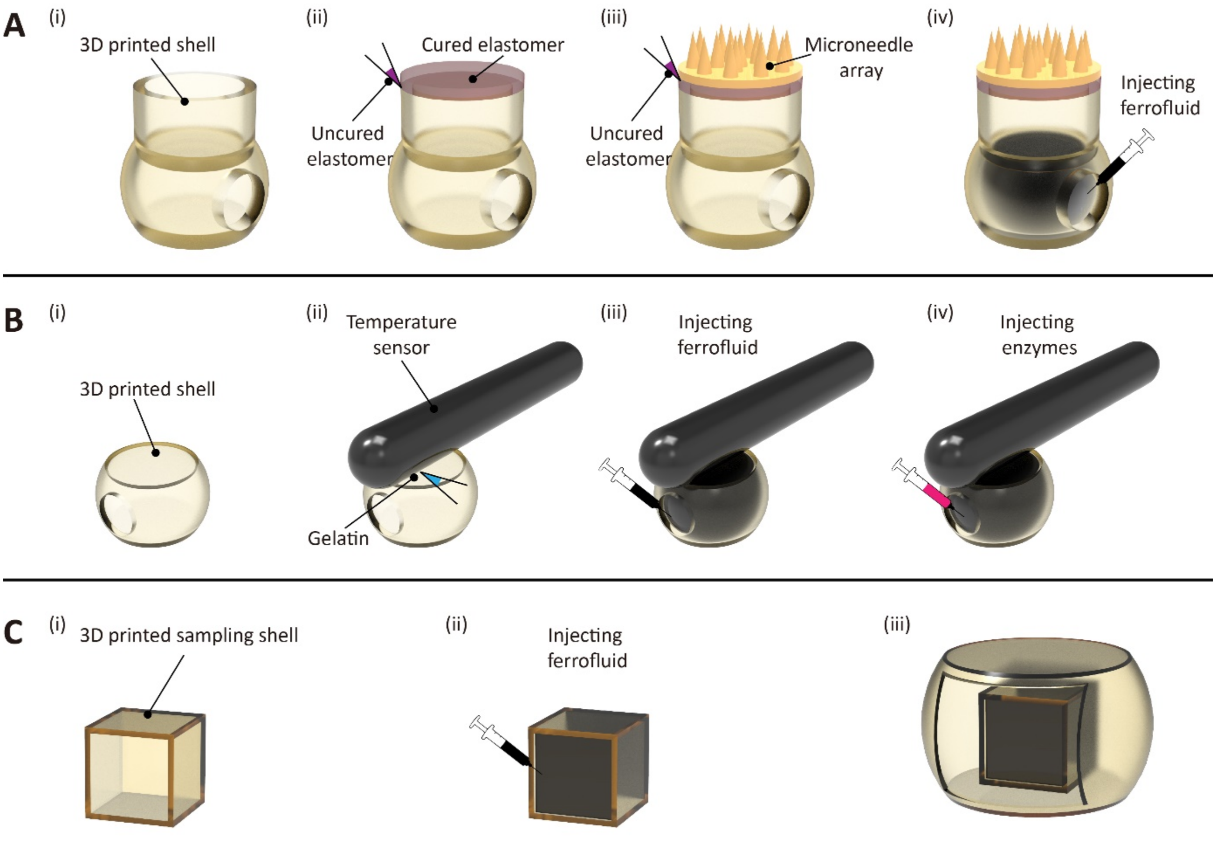

Fig. S16. Schematics of the fabrication process of the needle, sensor, and sampling robots' body. A. Illustration of fabricating the needle robot. The 3D-printed shell, the mouth of which is first sealed with an elastomer sheet, is then injected with a low-boiling-point fluid into the interior, then the 3D-printed microneedle array is attached to the surface of the elastomer sheet, and finally the ferrofluid is injected into the cavity to form the microneedle robot. **B.** Illustration of fabricating the sensor robot. The temperature sensor was first bonded to the 3D printed shell with gelatin, then ferrofluid was injected into the cavity, and finally an enzyme that can dissolve gelatin was injected into the ferrofluid. **C.** Illustration of fabricating the sampling robot. The sampled cavity is first filled with ferrofluid to seal it, and then it is placed into the larger shell.

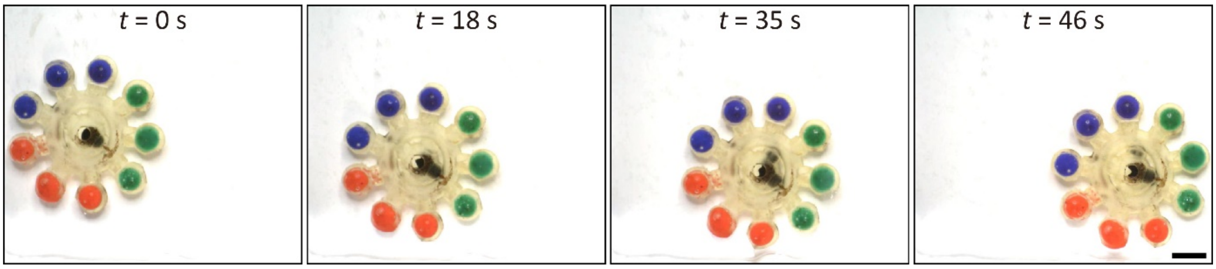

Fig. S17. The circular configuration is driven by a magnetic field for directional motion. Scale bar, 3 mm.

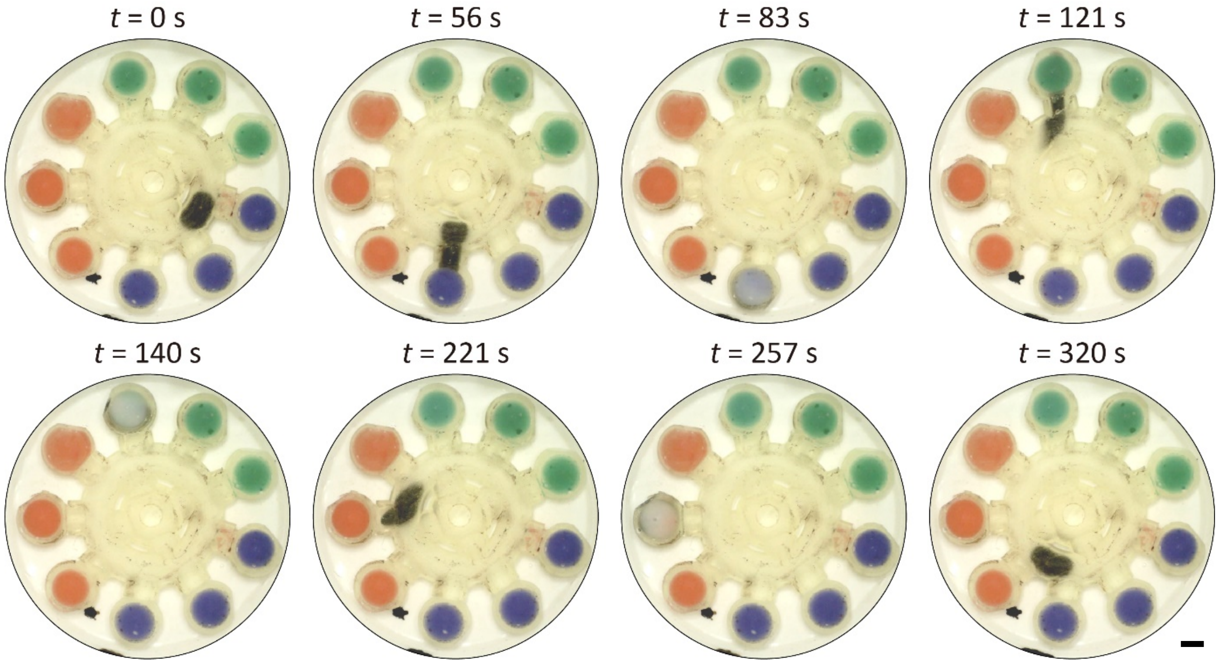

Fig. S18. Different functional analog areas can be sequentially triggered to change color within the circular configuration of the ferrofluid. Scale bar, 1 mm.

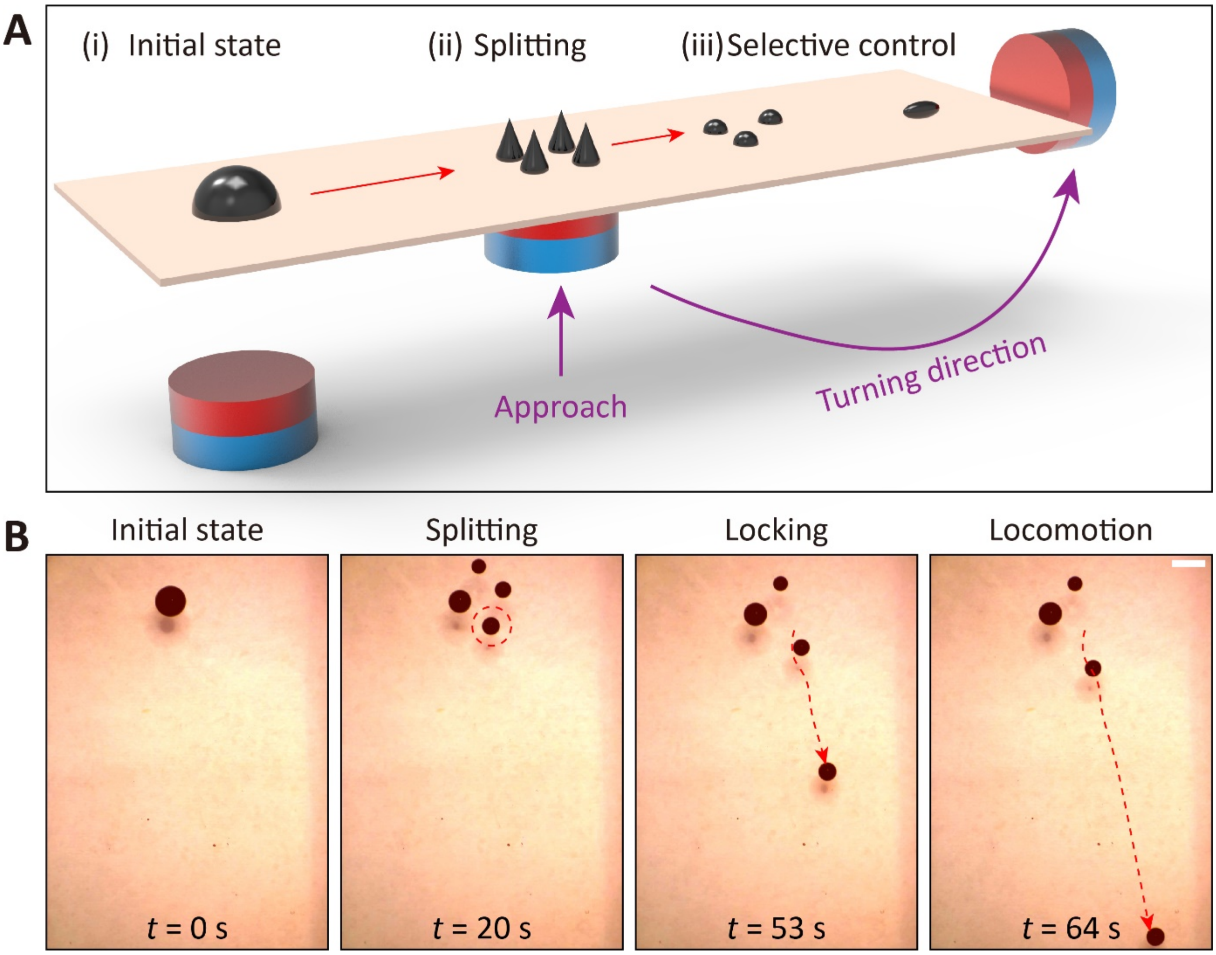

Fig. S19. Selective control strategy of ferrofluid droplets. A. Schematic diagram of the selective control strategy of ferrofluid droplets. First, a permanent magnet is used close to the ferrofluid to split it, and then the direction and position of the permanent magnet is reversed to attract the target sub-droplet for its individual movement. **B.** The image sequence shows the experimental process of the selective control of a ferrofluid droplet. Scale bar, 3 mm.

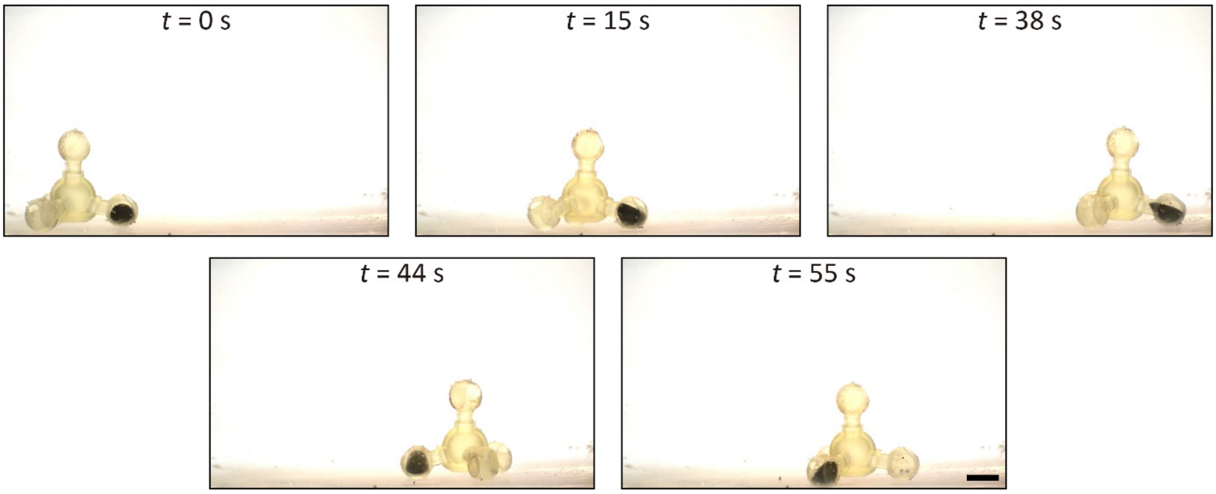

Fig. S20. Three-dimensional configurations undergo directional motion driven by a magnetic field. Scale bar, 3 mm.

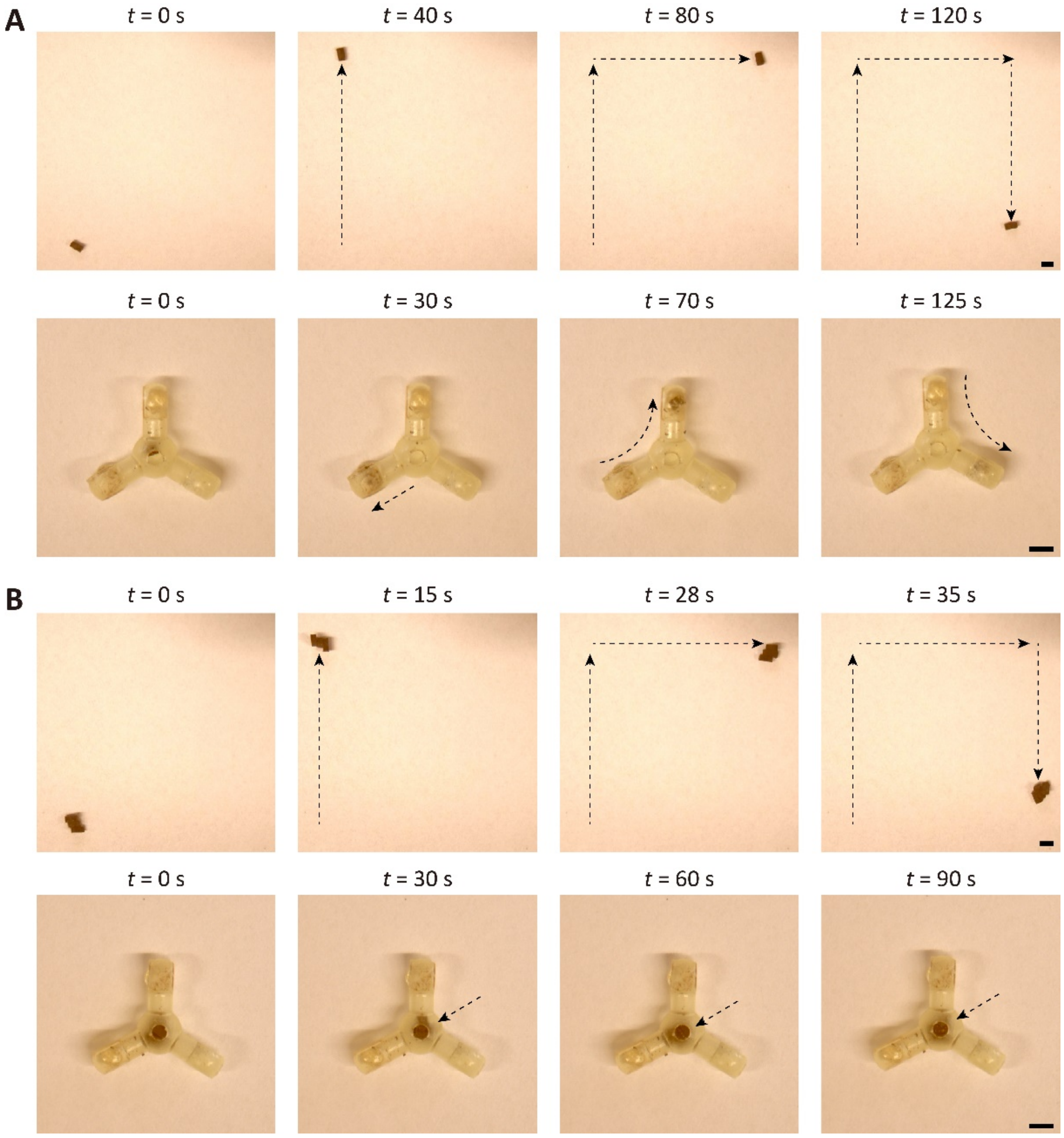

Fig. S21. Design of a magnetic soft robot consisting of hard magnetic particles combined with a solid shell. A. Controlled motion of a single magnetic soft robot composed of hard magnetic particles in a plane and in a solid shell. **B.** Controlled motion of aggregates formed by magnetic soft robots composed of hard magnetic particles in the plane and in the solid shells. This magnetic soft robot can perform controlled motions, and when combined with a solid shell, they can sequentially enter the desired spatial region, which means that it is expected that different functional modules can be triggered sequentially. However, to achieve simultaneous triggering of multiple functional modules, the number of soft-body robots needs to be increased since magnetic elastomer robots cannot be split, e.g., three magnetic soft-body robots are needed to trigger three functional modules at the same time. But when the three magnetic soft robots come into contact, they aggregate together and although they can move in a controlled manner, controlled disassembly cannot be realized. This means that it is not possible to realize the triggering of multiple functions at the same time. Scale bars, 4 mm.

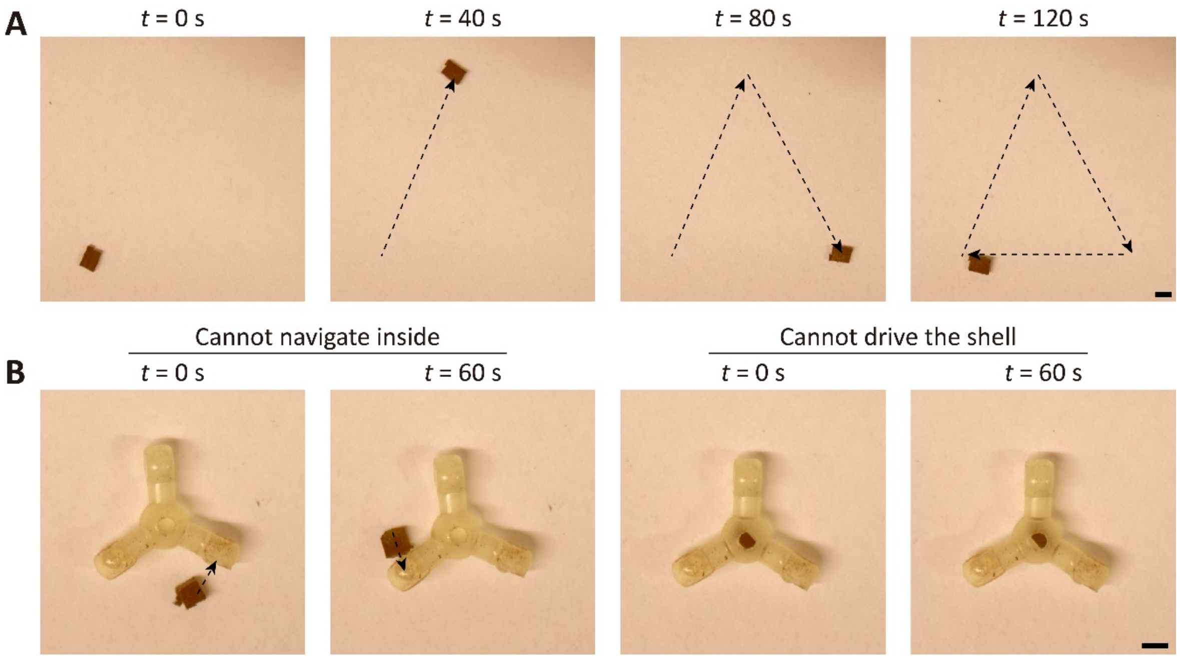

Fig. S22. Design of a magnetic soft robot composed of soft magnetic particles combined with a solid shell. A. Controlled motion of a single magnetic soft robot composed of soft magnetic particles in a plane. **B.** Controlled motion of single and collective magnetic soft robots composed of soft magnetic particles in the solid shells. Although the monoliths can also perform controlled movements, the magnetic soft body robots are not able to enter the interior of the solid shell due to their inability to perform extreme deformations. In our work, the droplet can pass through the deformation into a hole much smaller than its own size. To control the entry of a magnetic soft body robot, its size would need to be reduced. This would significantly affect its actuation and magneto-thermal properties. In addition, for multiple magneto-soft robots combined with a solid shell, it is also not possible to effectively separate them in order to trigger multiple functional modules at the same time. Scale bars, 4 mm.

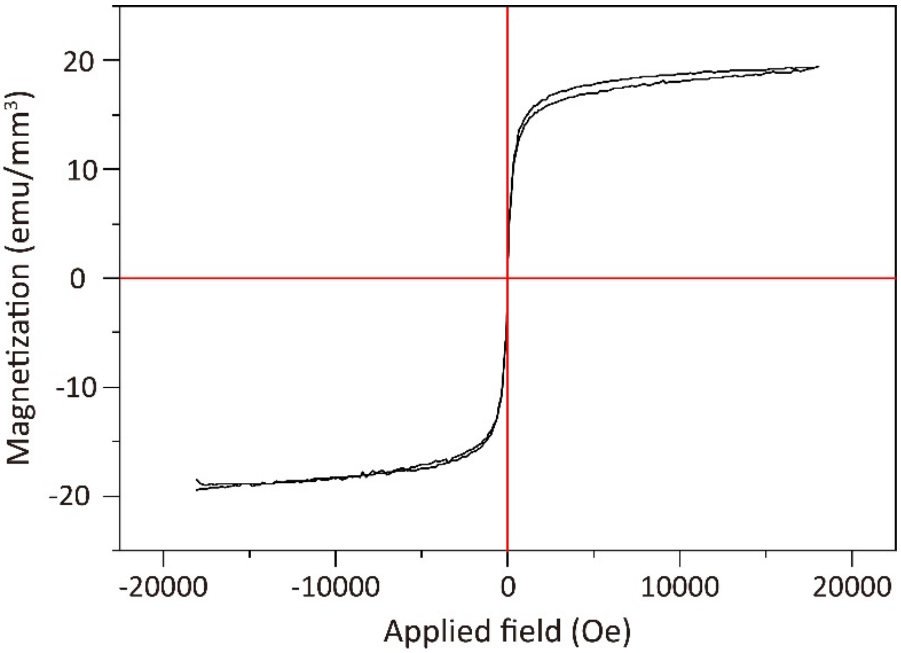

Fig. S23. Magnetic hysteresis loops for ferrofluid. We use the vibrating sample magnetometer (EZ7 VSM, MicroSense LLC) to measure the magnetization properties of the ferrofluid and its saturation magnetization strength is 270 mT. This value is different from the value of 44 mT provided by the manufacturer when the ferrofluid was purchased, thus we changed the value to 270 mT in the revised manuscript. The saturation magnetization strength is when the material is magnetized to reach the maximum possible magnetization with the application of an external magnetic field. When all the magnetic particles or atoms in the material are magnetized and no further increase in magnetization is possible, the material reaches a state of magnetic saturation. In the magnetically saturated state, the magnetization of the material remains constant regardless of the continued increase in the external magnetic field. The plot shows that the required magnetic field strength needed to reach saturation is around 270 mT (2700 Oe), beyond which the hysteresis curve plateaus. In this regard, the morphology of the ferrofluid droplet under magnetic field with strength up to 160 mT is controllable and repeatable. In addition, the ferrofluid remains stable even when the external magnetic field strength exceeds 270 mT. Although the ferrofluid droplets are magnetically saturated, the ferromagnetic particles inside the ferrofluid are encapsulated by the surfactant to prevent them from coalescing due to van der Waals forces and magnetic forces. In performing the hysteresis curve test, the machine reached a maximum strength of 1.8 T, and the droplets could still be driven in a controlled manner after the test.

Table S1. Composition, physical and chemical properties of ferrofluid.

**Appearance:** Black fluid	**Viscosity @27°C:** 8 mPa·s
**Iron Oxide (magnetite):** 9% (by volume)	**Density @25°C:** 1.49 g/cc
**Oleic Acid Dispersant:** 18% (by volume)	**Pour Point:** -94 °C
**Distillates (Petroleum) Hydrotreated Light:** 73% (by volume)	**Flash Point:** 89 °C
**Nominal Particle Diameter:** 10 nm	**Initial Magnetic Susceptibility:** 0.28
**Saturation Magnetization:** 270 mT	**Water Solubility:** Insoluble

Supplementary Movies

Movie S1. Mechanisms for assembling, actuating, and separating ferrofluids with solid shells of different sizes
Movie S2. Selective assembly of ferrofluid droplets with different solid shells
Movie S3. Magnetic sub-millimeter scale solid-droplet system for cell transplantation
Movie S4. Magnetic millimeter scale solid-droplet system for stent delivery
Movie S5. Magnetic millimeter scale solid-droplet system serves as microneedles patch
Movie S6. Magnetic millimeter scale solid-droplet system serves as sensor delivery robot
Movie S7. Magnetic millimeter scale solid-droplet system serves as liquid sampling robot
Movie S8. Multifunctional magnetic solid-droplet system with decoupling capability
Movie S9. Magnetic solid-droplet system with different configurations
